# Supplementary material for: Topological data analysis of spatial patterning in heterogeneous cell populations: clustering and sorting with varying cell-cell adhesion
Source: NPJ Syst Biol Appl. 2023 Sep 14;9:43. doi: 10.1038/s41540-023-00302-8 (PMC10502054; doi:10.1038/s41540-023-00302-8)
Supplement: Supplementary file 1 — Supplementary Information [file 41540_2023_302_MOESM1_ESM.pdf]

Supplementary Information

"Topological Data Analysis of Spatial Patterning in Heterogeneous Cell Populations: Clustering and Sorting with Varying Cell-Cell Adhesion"

D. Bhaskar, W.Y. Zhang, A. Volkening, B. Sandstede, and I.Y. Wong

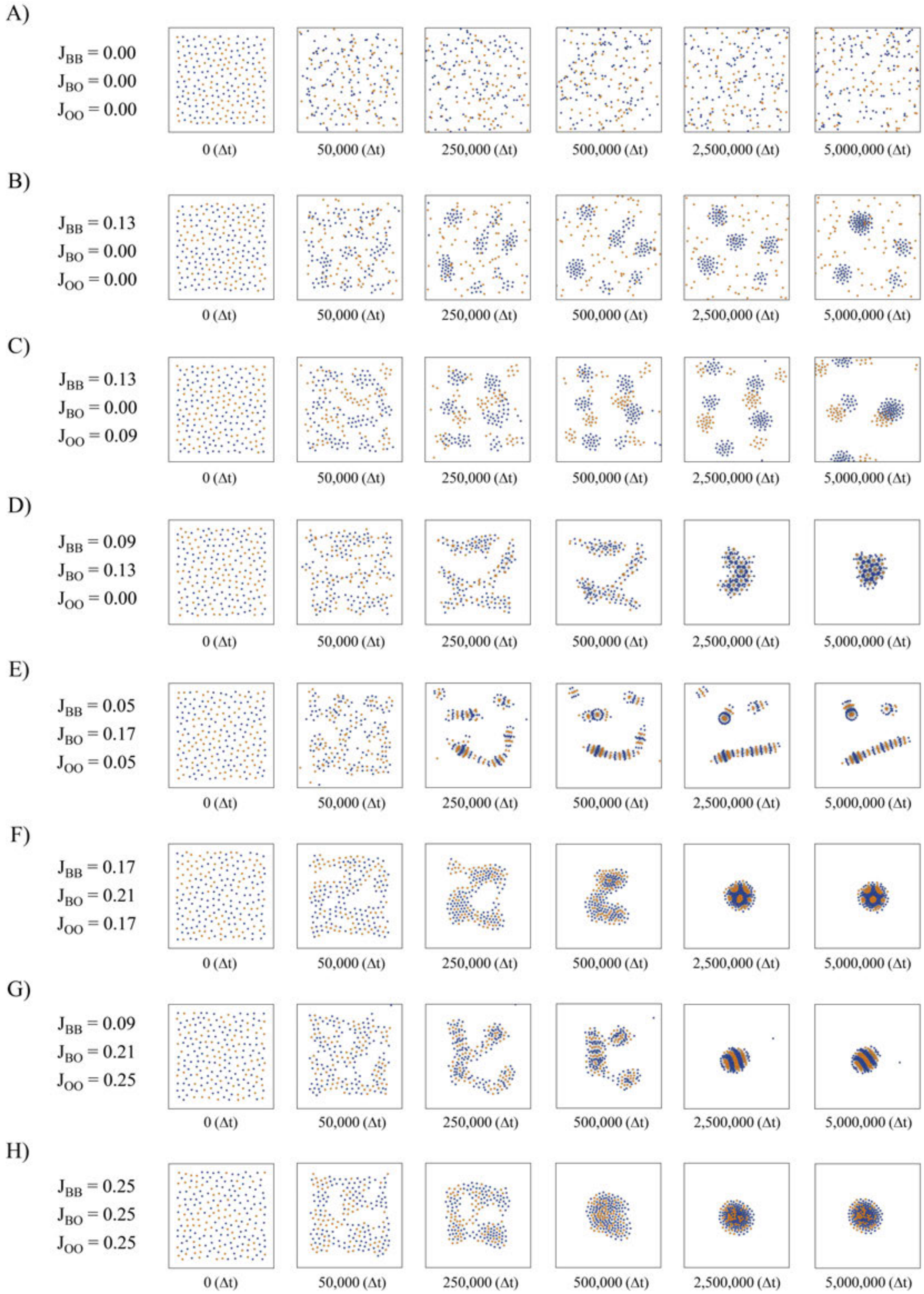

Supplementary Figure 1: **Simulation snapshots showing self-organization in a heterogeneous population over time (log scale) at varying differential adhesion parameters.** (A) Particles remain individually dispersed at low adhesion values. (B) Blue particles aggregate into clusters due to high blue-blue adhesion. (C) Complete sorting simulation where both blue and orange particles form separate clusters due to high homotypic adhesion. (D-G) High heterotypic adhesion results in configurations that maximize the interaction between the two cell types, forming hexagonal, striped and spotted patterns. (H) Well-mixed clusters are obtained when homotypic and heterotypic adhesion values are greater than zero and (approximately) equal.

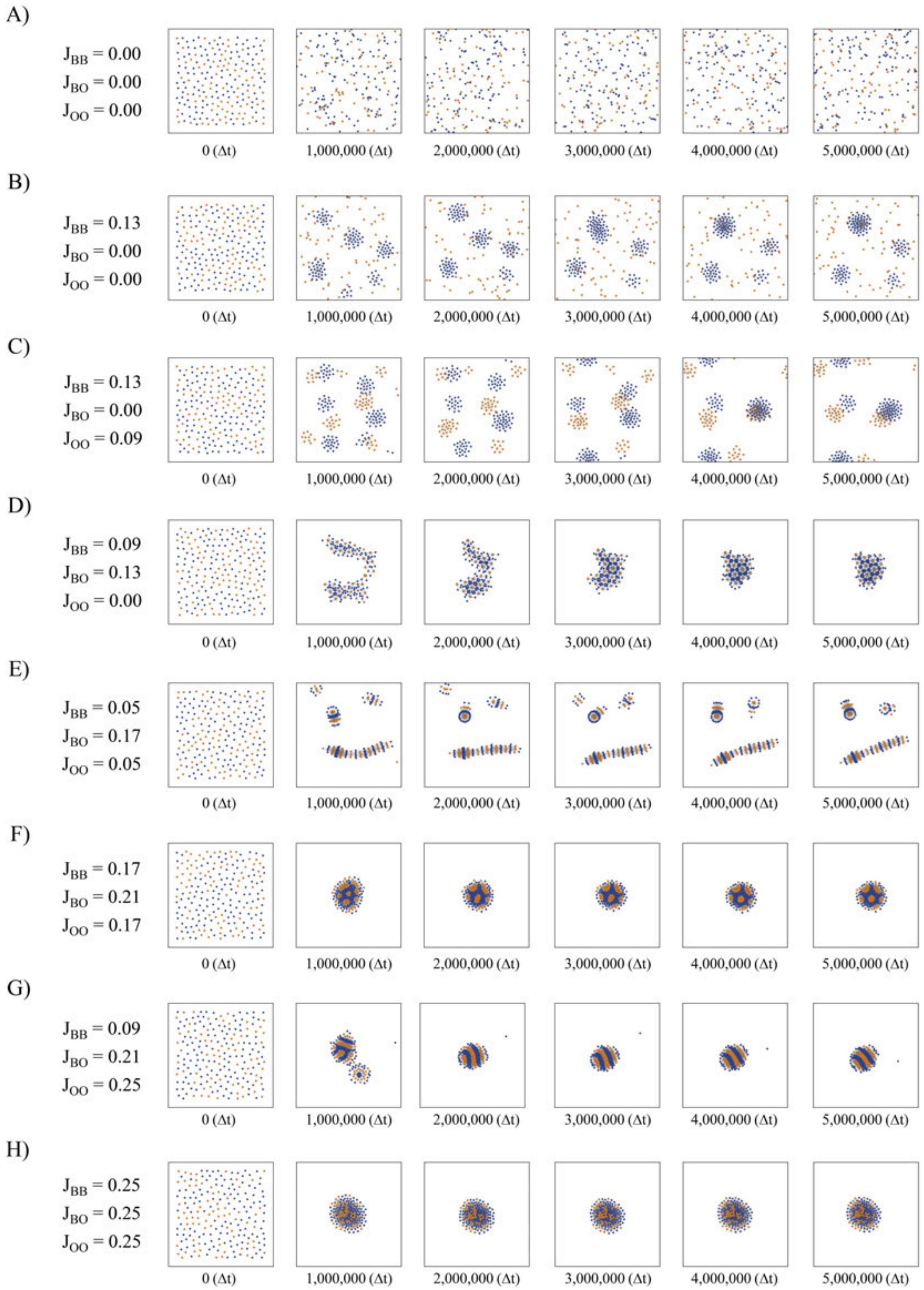

Supplementary Figure 2: **Simulation snapshots showing self-organization in a heterogeneous population over time (linear scale) at varying differential adhesion parameters.** (A) Particles remain individually dispersed at low adhesion values. (B) Blue particles aggregate into clusters due to high blue-blue adhesion. (C) Complete sorting simulation where both blue and orange particles form separate clusters due to high homotypic adhesion. (D-G) High heterotypic adhesion results in configurations that maximize the interaction between the two cell types, forming hexagonal, striped and spotted patterns. (H) Well-mixed clusters are obtained when homotypic and heterotypic adhesion values are greater than zero and (approximately) equal.

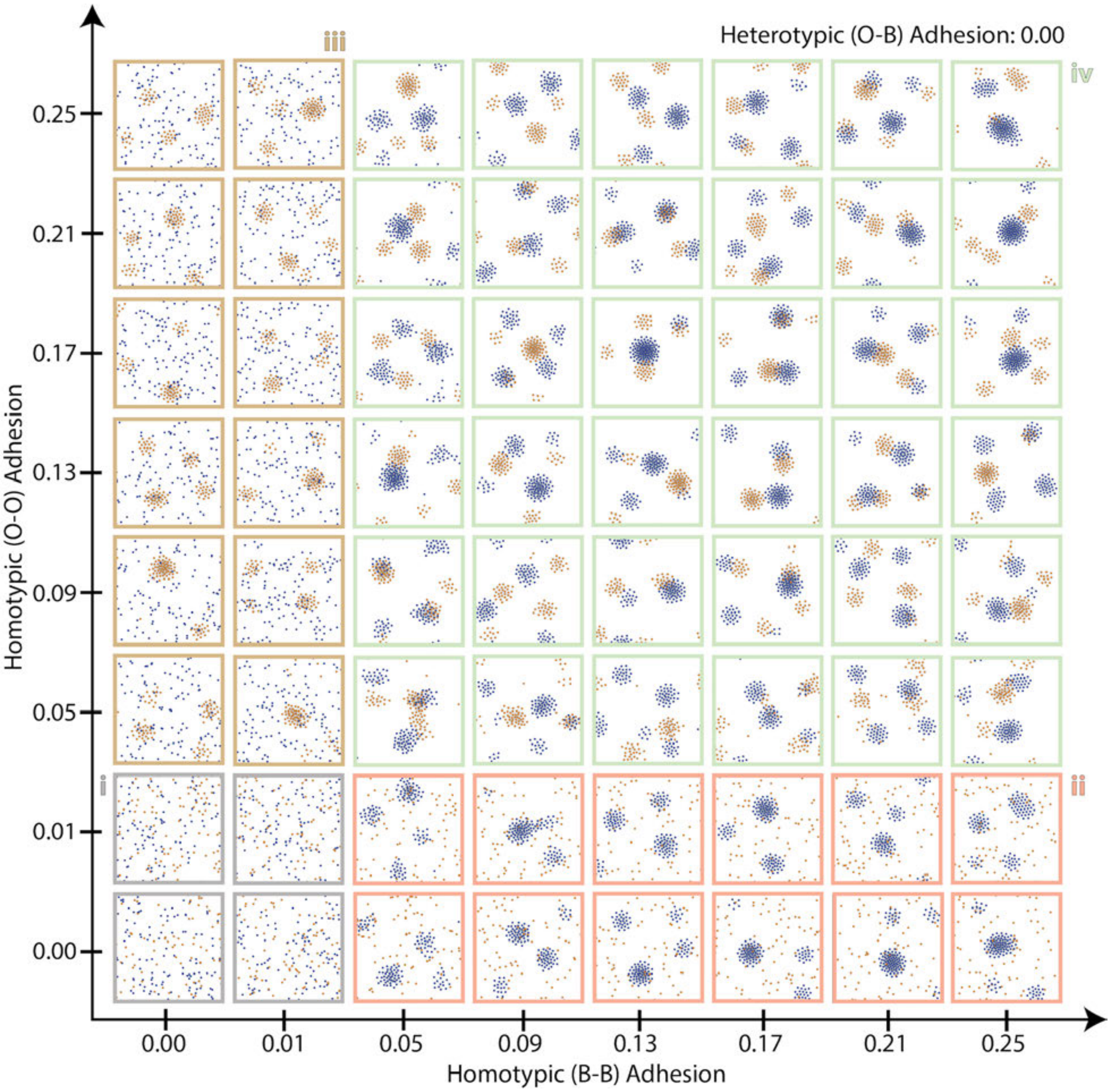

Supplementary Figure 3: **Simulation snapshots of multicellular patterning of a heterogeneous, non-proliferating population at steady state with $\mathbf{J_{BO} = 0.00}$ and varying $\mathbf{J_{BB}, J_{OO}}$.** i. denotes individually dispersed blue and orange cells. ii denotes clusters of blue cells with individually dispersed orange cells. iii denotes individually dispersed blue cells with clusters of orange cells. iv. denotes separate clusters of orange and blue cells, respectively.

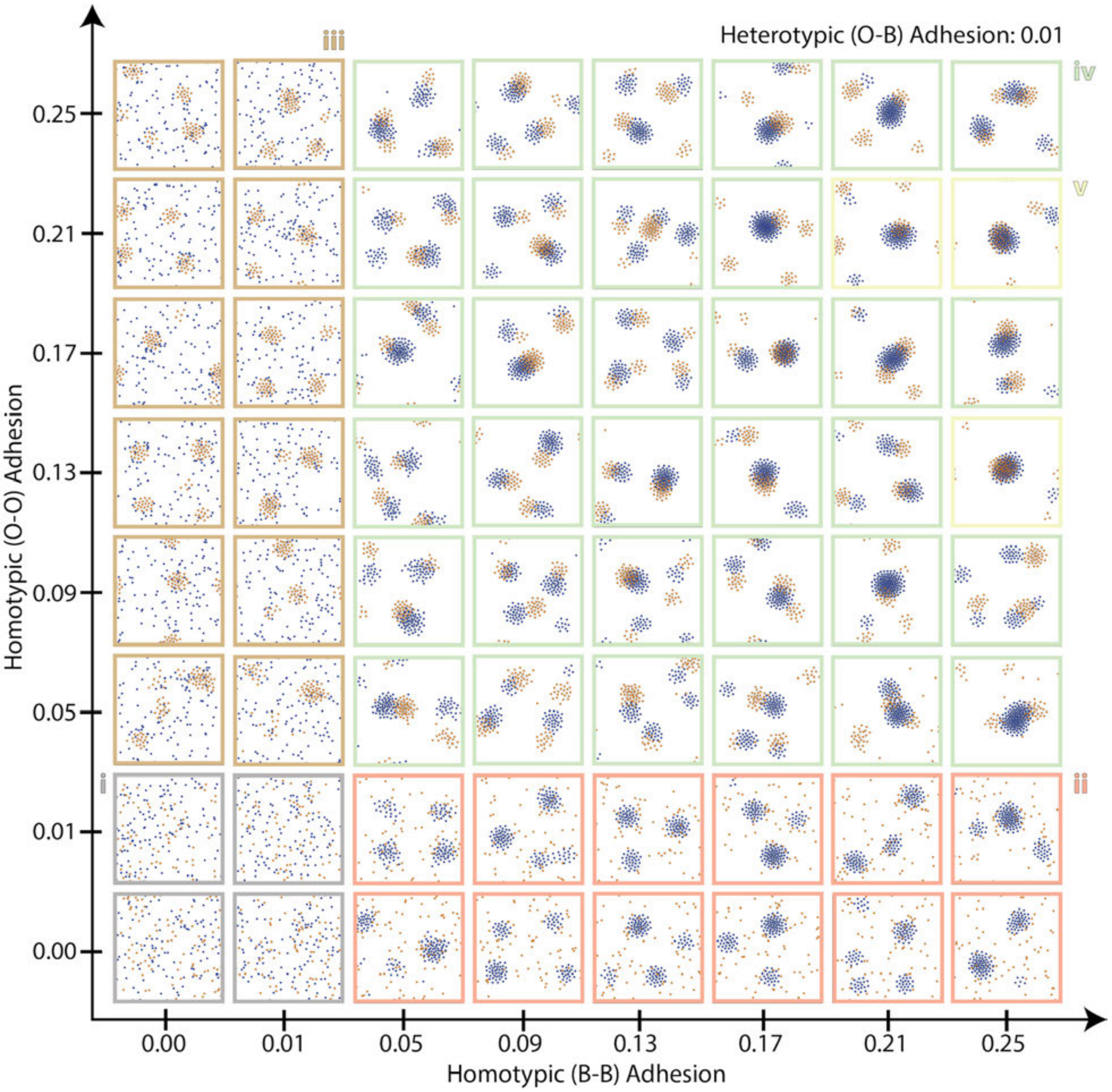

Supplementary Figure 4: **Simulation snapshots of multicellular patterning of a heterogeneous, non-proliferating population at steady state with $\mathbf{J_{BO} = 0.01}$ and varying $\mathbf{J_{BB}, J_{OO}}$.** i. denotes individually dispersed blue and orange cells. ii denotes clusters of blue cells with individually dispersed orange cells. iii denotes individually dispersed blue cells with clusters of orange cells. iv. denotes separate clusters of orange and blue cells, respectively. v denotes clusters with intermixed blue and orange cells.

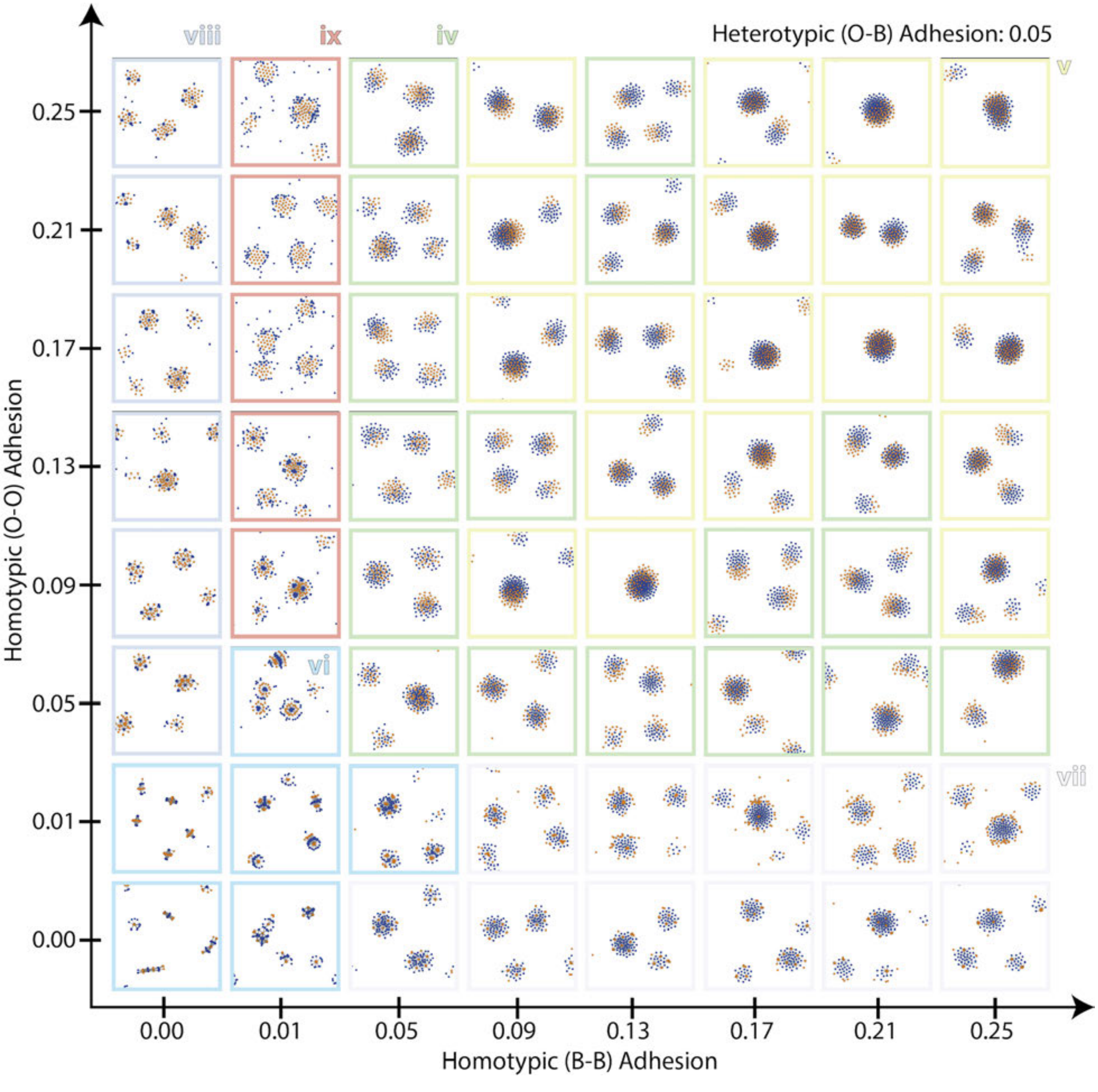

Supplementary Figure 5: **Simulation snapshots of multicellular patterning of a heterogeneous, non-proliferating population at steady state with $\mathbf{J_{BO} = 0.05}$ and varying $\mathbf{J_{BB}, J_{OO}}$.** iv. denotes separate clusters of orange and blue cells, respectively. v denotes clusters with intermixed blue and orange cells. vi denotes clusters with alternating stripes of blue and orange cells. vii denotes hexagonal arrays of orange cells surrounded by blue cells. viii denotes hexagonal arrays of blue cells surrounded by orange cells. ix denotes a cluster with a core of orange cells surrounded by blue cells.

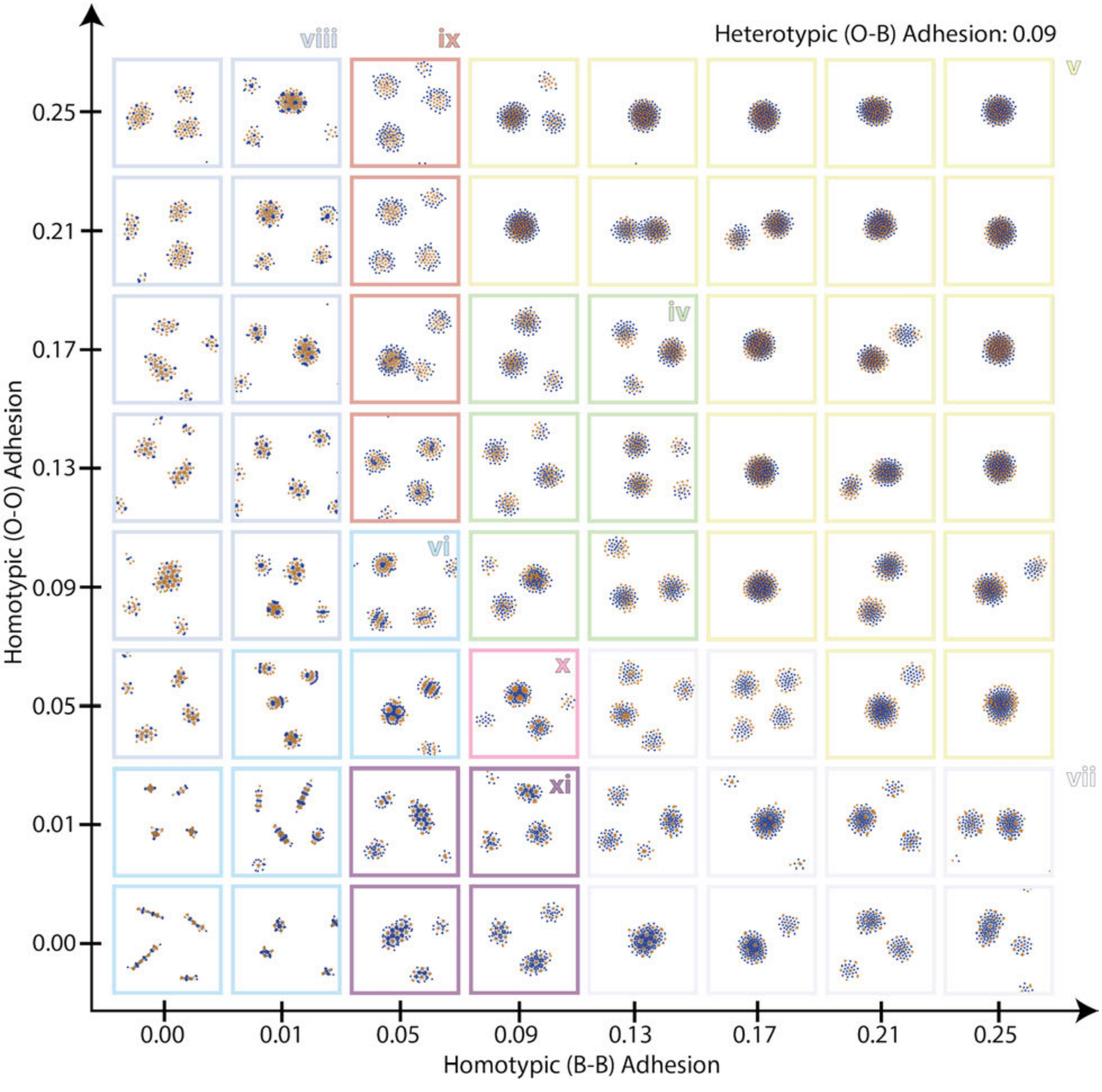

Supplementary Figure 6: **Simulation snapshots of multicellular patterning of a heterogeneous, non-proliferating population at steady state with $\mathbf{J_{BO} = 0.09}$ and varying $\mathbf{J_{BB}, J_{OO}}$.** iv. denotes separate clusters of orange and blue cells, respectively. v denotes clusters with intermixed blue and orange cells. vi denotes clusters with alternating stripes of blue and orange cells. vii denotes hexagonal arrays of orange cells surrounded by blue cells. viii denotes hexagonal arrays of blue cells surrounded by orange cells. ix denotes a cluster with a core of orange cells surrounded by blue cells.

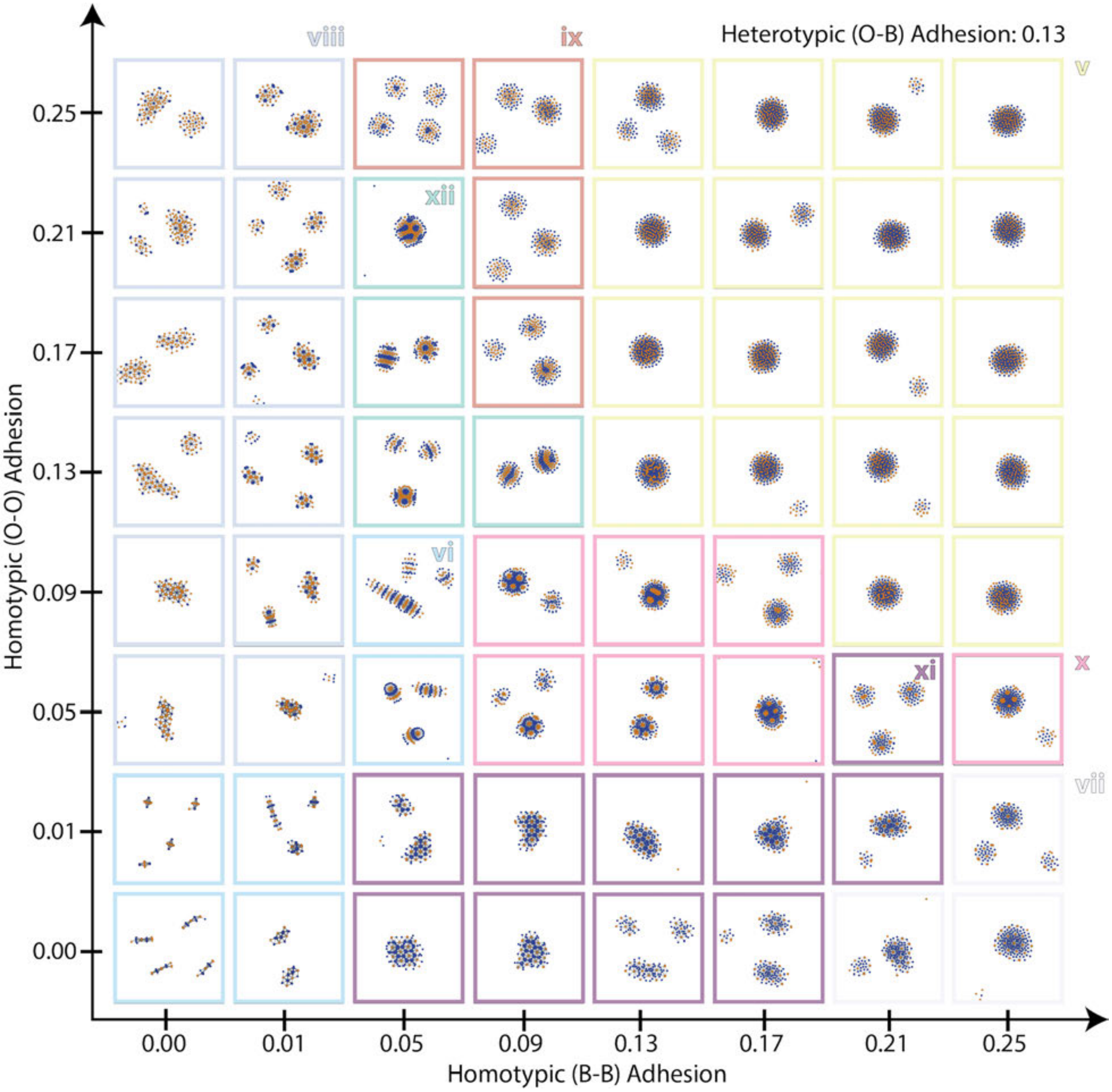

Supplementary Figure 7: **Simulation snapshots of multicellular patterning of a heterogeneous, non-proliferating population at steady state with $\mathbf{J_{BO} = 0.13}$ and varying $\mathbf{J_{BB}, J_{OO}}$.** v denotes clusters with intermixed blue and orange cells. vi denotes clusters with alternating stripes of blue and orange cells. vii denotes hexagonal arrays of orange cells surrounded by blue cells. viii denotes hexagonal arrays of blue cells surrounded by orange cells. ix denotes a cluster with a core of orange cells surrounded by blue cells. x denotes tightly packed hexagonal arrays of orange cells surrounded by tightly packed blue cells. xi denotes hexagonal arrays of orange cells surrounded by tightly packed blue cells. xii denotes labyrinth patterns of blue and orange cells.

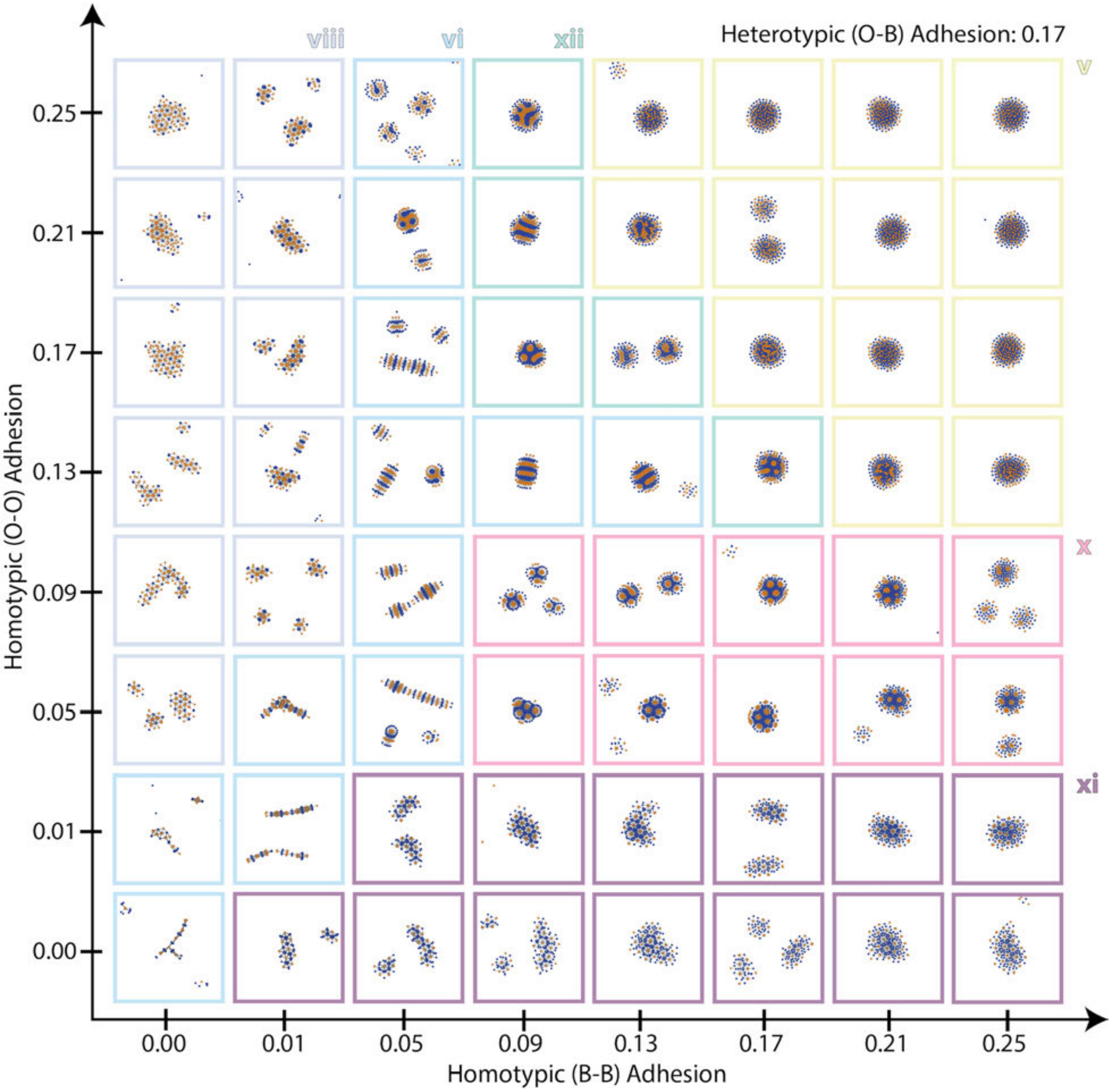

Supplementary Figure 8: **Simulation snapshots of multicellular patterning of a heterogeneous, non-proliferating population at steady state with $\mathbf{J_{BO} = 0.17}$ and varying $\mathbf{J_{BB}, J_{OO}}$.** v denotes clusters with intermixed blue and orange cells. vi denotes clusters with alternating stripes of blue and orange cells. vii denotes hexagonal arrays of orange cells surrounded by blue cells. viii denotes hexagonal arrays of blue cells surrounded by orange cells. ix denotes a cluster with a core of orange cells surrounded by blue cells. x denotes tightly packed hexagonal arrays of orange cells surrounded by tightly packed blue cells. xi denotes hexagonal arrays of orange cells surrounded by tightly packed blue cells. xii denotes labyrinth patterns of blue and orange cells.

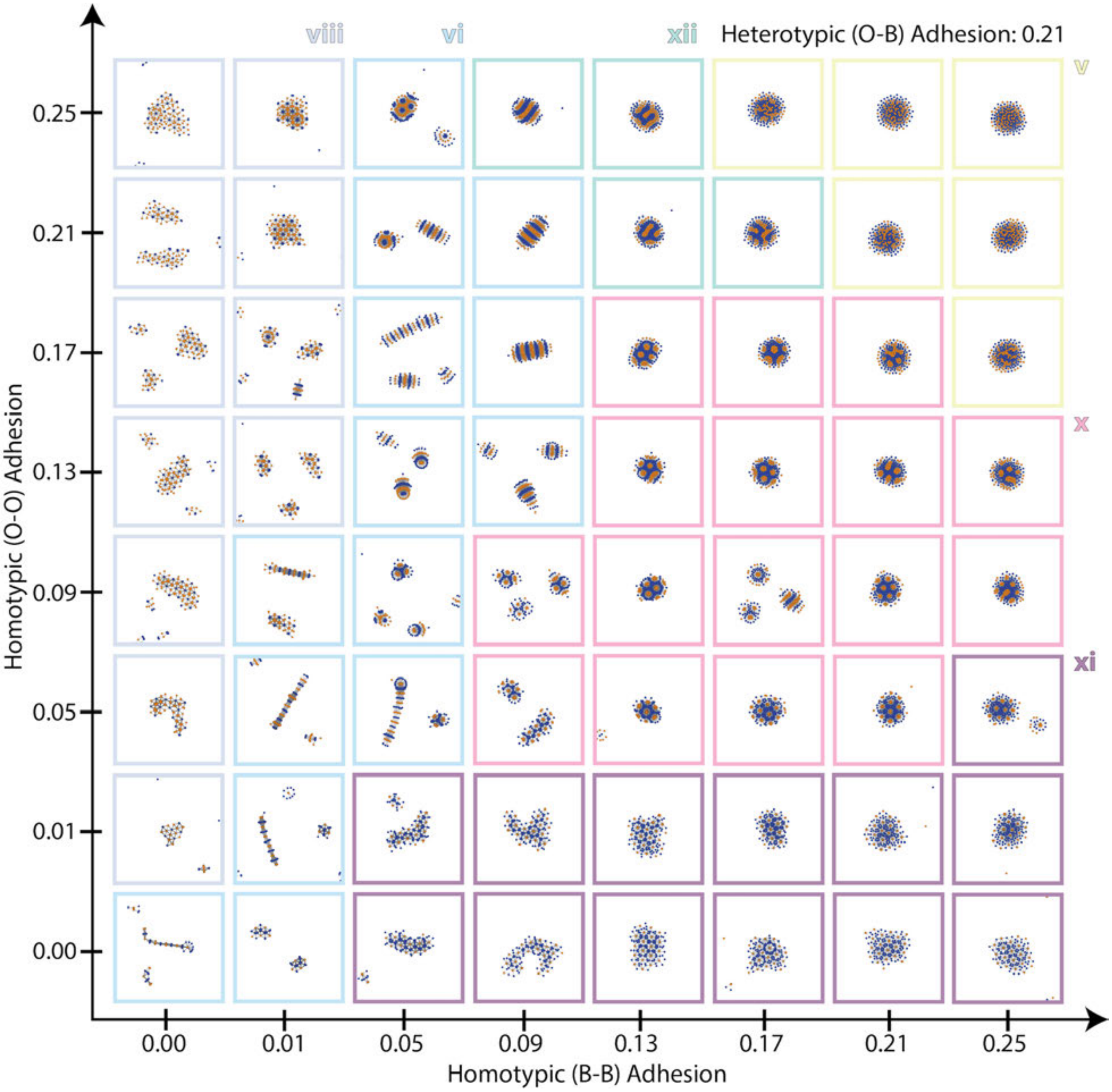

Supplementary Figure 9: **Simulation snapshots of multicellular patterning of a heterogeneous, non-proliferating population at steady state with $\mathbf{J_{BO} = 0.21}$ and varying $\mathbf{J_{BB}, J_{OO}}$.** v denotes clusters with intermixed blue and orange cells. vi denotes clusters with alternating stripes of blue and orange cells. vii denotes hexagonal arrays of orange cells surrounded by blue cells. viii denotes hexagonal arrays of blue cells surrounded by orange cells. ix denotes a cluster with a core of orange cells surrounded by blue cells. x denotes tightly packed hexagonal arrays of orange cells surrounded by tightly packed blue cells. xi denotes hexagonal arrays of orange cells surrounded by tightly packed blue cells. xii denotes labyrinth patterns of blue and orange cells.

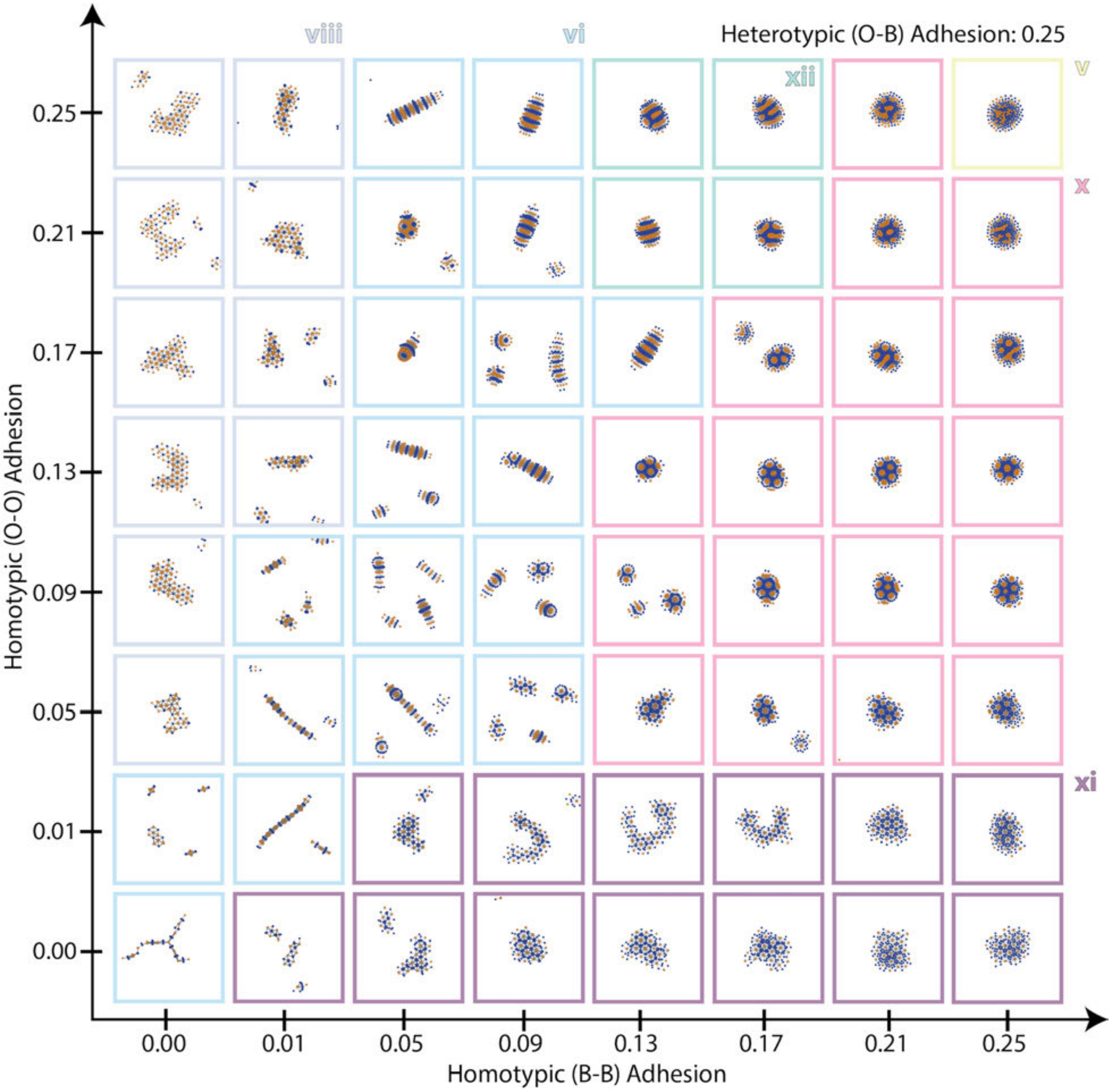

Supplementary Figure 10: **Simulation snapshots of multicellular patterning of a heterogeneous, non-proliferating population at steady state with $\mathbf{J_{BO} = 0.25}$ and varying $\mathbf{J_{BB}, J_{OO}}$.** v denotes clusters with intermixed blue and orange cells. vi denotes clusters with alternating stripes of blue and orange cells. viii denotes hexagonal arrays of blue cells surrounded by orange cells. x denotes tightly packed hexagonal arrays of orange cells surrounded by tightly packed blue cells. xi denotes hexagonal arrays of orange cells surrounded by tightly packed blue cells. xii denotes labyrinth patterns of blue and orange cells.

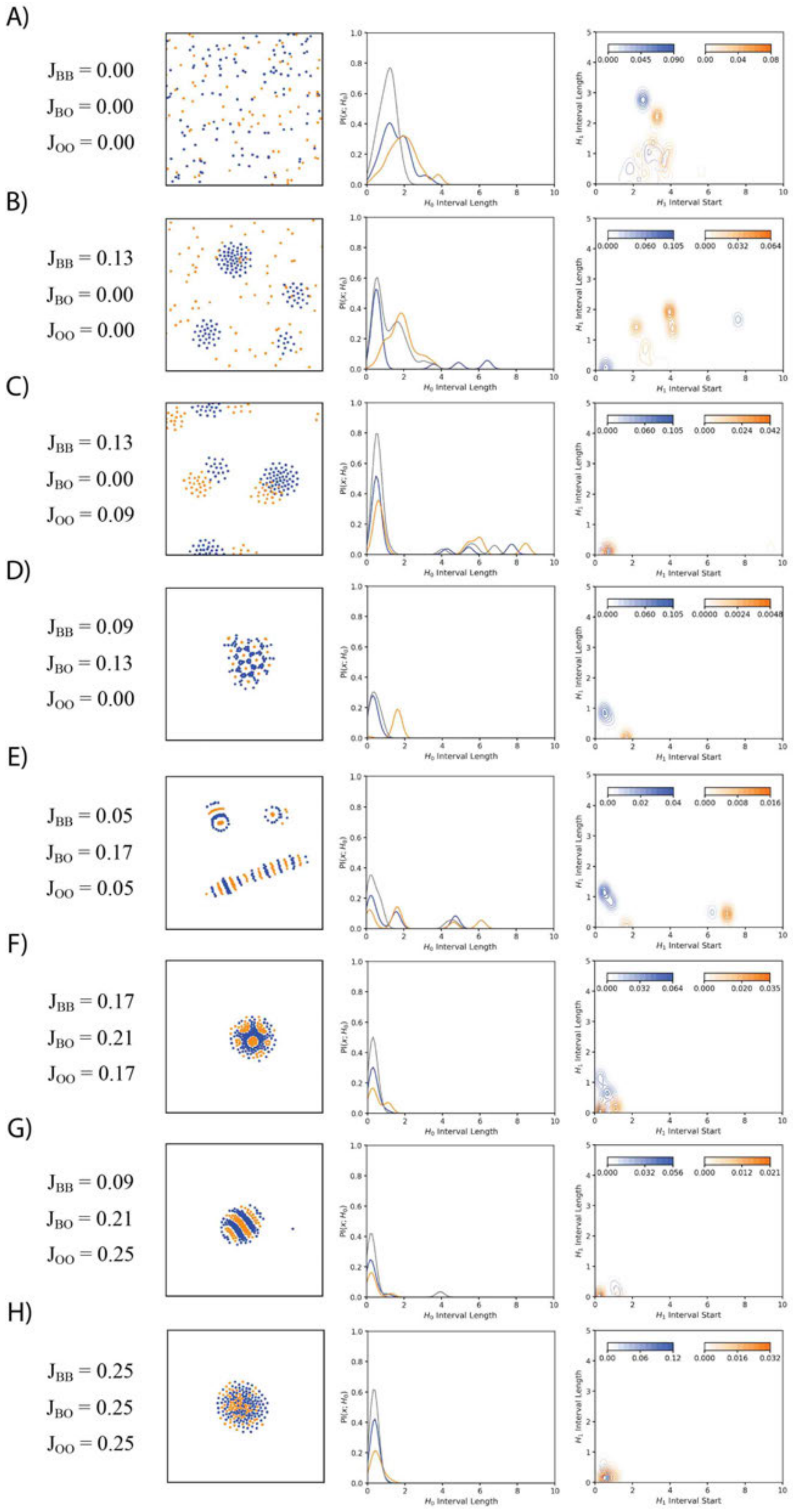

Supplementary Figure 11: **Persistence images for representative particle configurations.** Adhesion parameter values are provided in the first column. Particle positions for the two cell types are plotted in blue and orange at the final time-step of the simulation in the second column. Intensity values for the dimension 0 persistence image is shown in the third column. Contour plots for dimension 1 persistence images are shown in the fourth column.

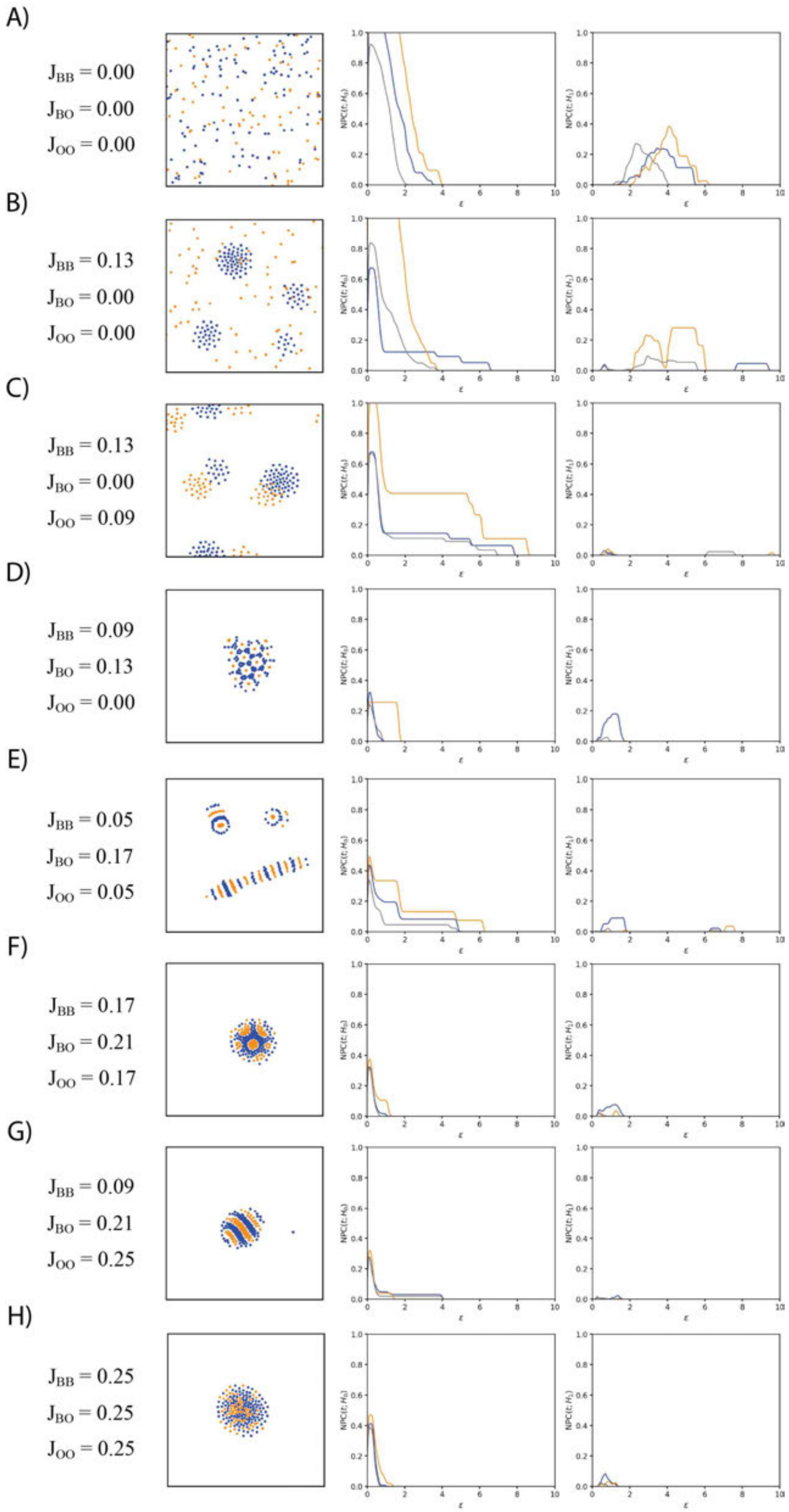

Supplementary Figure 12: **Normalized persistence curves for representative particle configurations.** Adhesion parameter values are provided in the first column. Particle positions for the two cell types are plotted in blue and orange at the final time-step of the simulation in the second column. Persistence curves corresponding to dimension 0 and 1 persistent homology are shown in the third and fourth columns respectively.

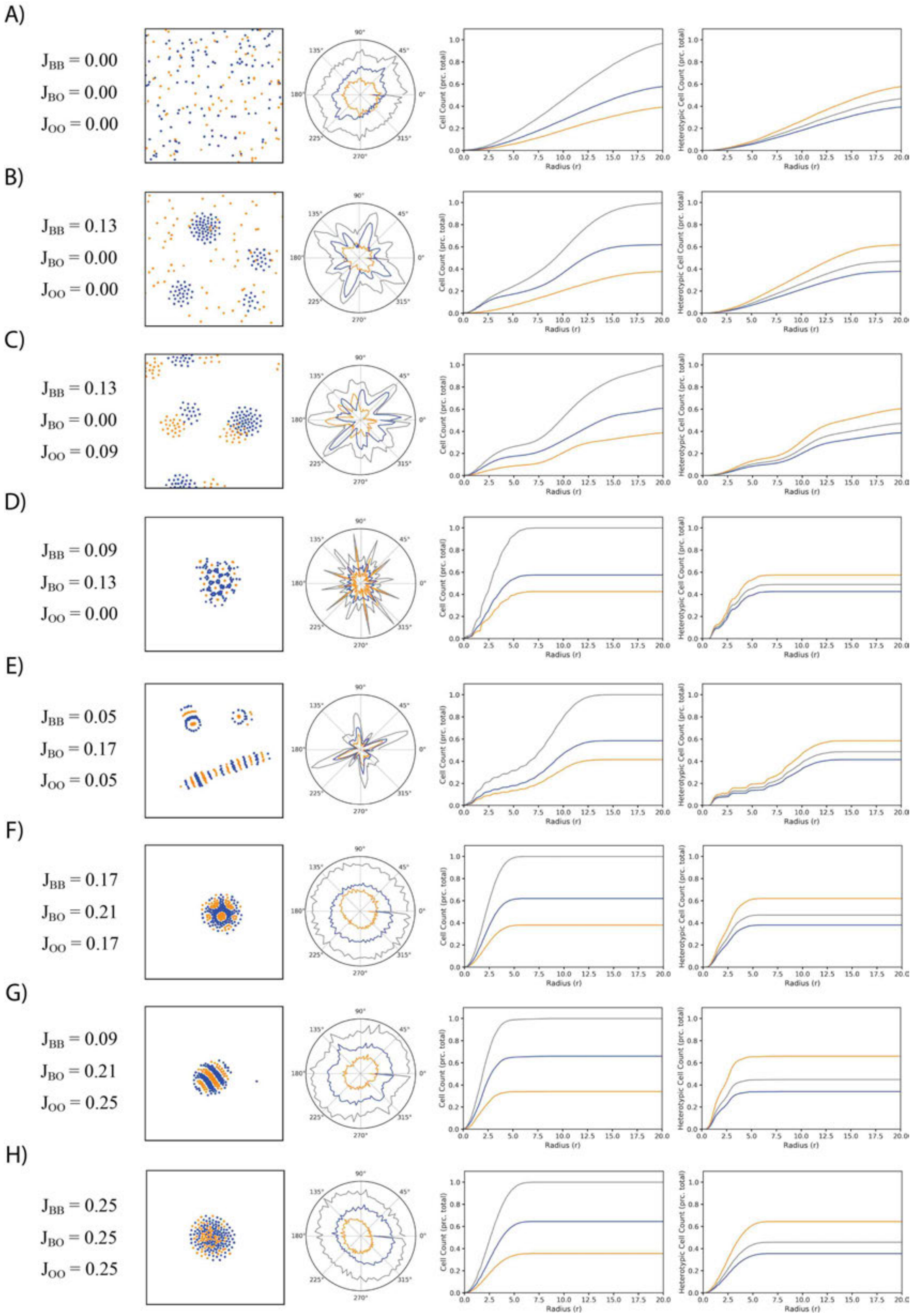

Supplementary Figure 13: **Order parameters for representative particle configurations.** Adhesion parameter values are provided in the first column. Particle positions for the two cell types are plotted in blue and orange at the final time-step of the simulation in the second column. Ensemble-averaged angular distribution, radial distribution for homotypic neighbors and radial distribution for heterotypic neighbors are shown in the third, fourth and fifth columns respectively.

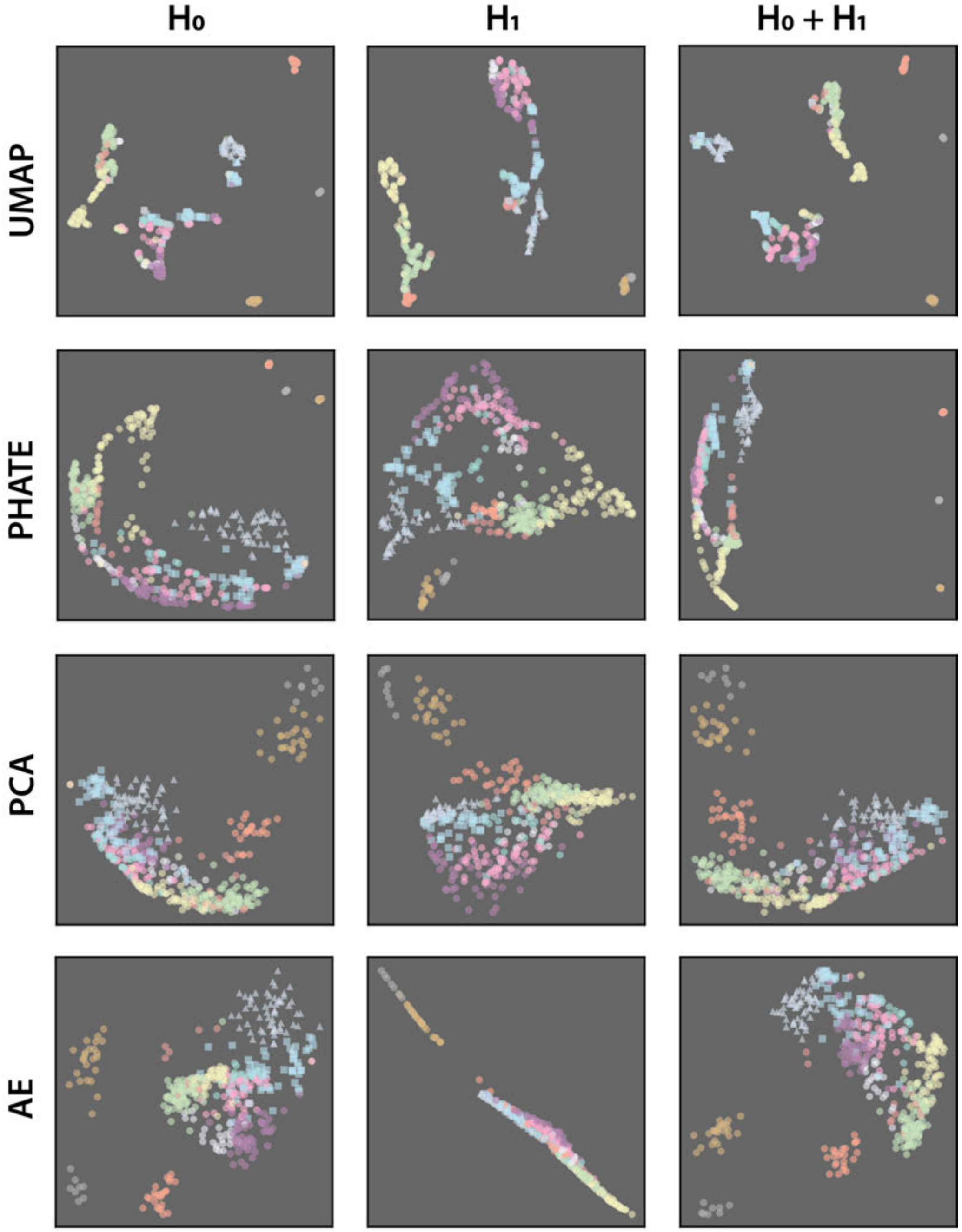

Supplementary Figure 14: **2D embeddings of persistence images of non-proliferating particle configurations.** Low dimension representation of simulated particle configurations obtained using UMAP, PHATE, and PCA. Embeddings computed using dimension 0, dimension 1, and concatenated persistence images for simulations with proliferation disabled.

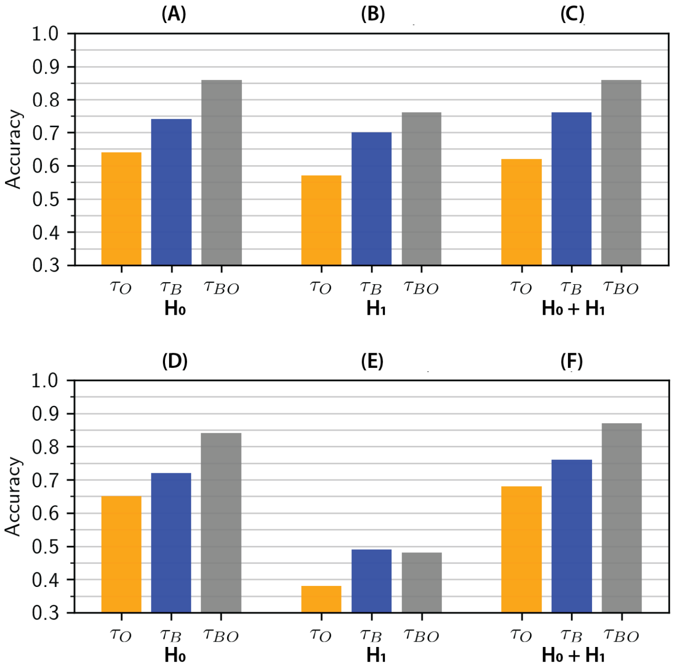

Supplementary Figure 15: **Classification accuracy of persistence curves.** (A-C) at constant population size. (D-F) at varying population size.

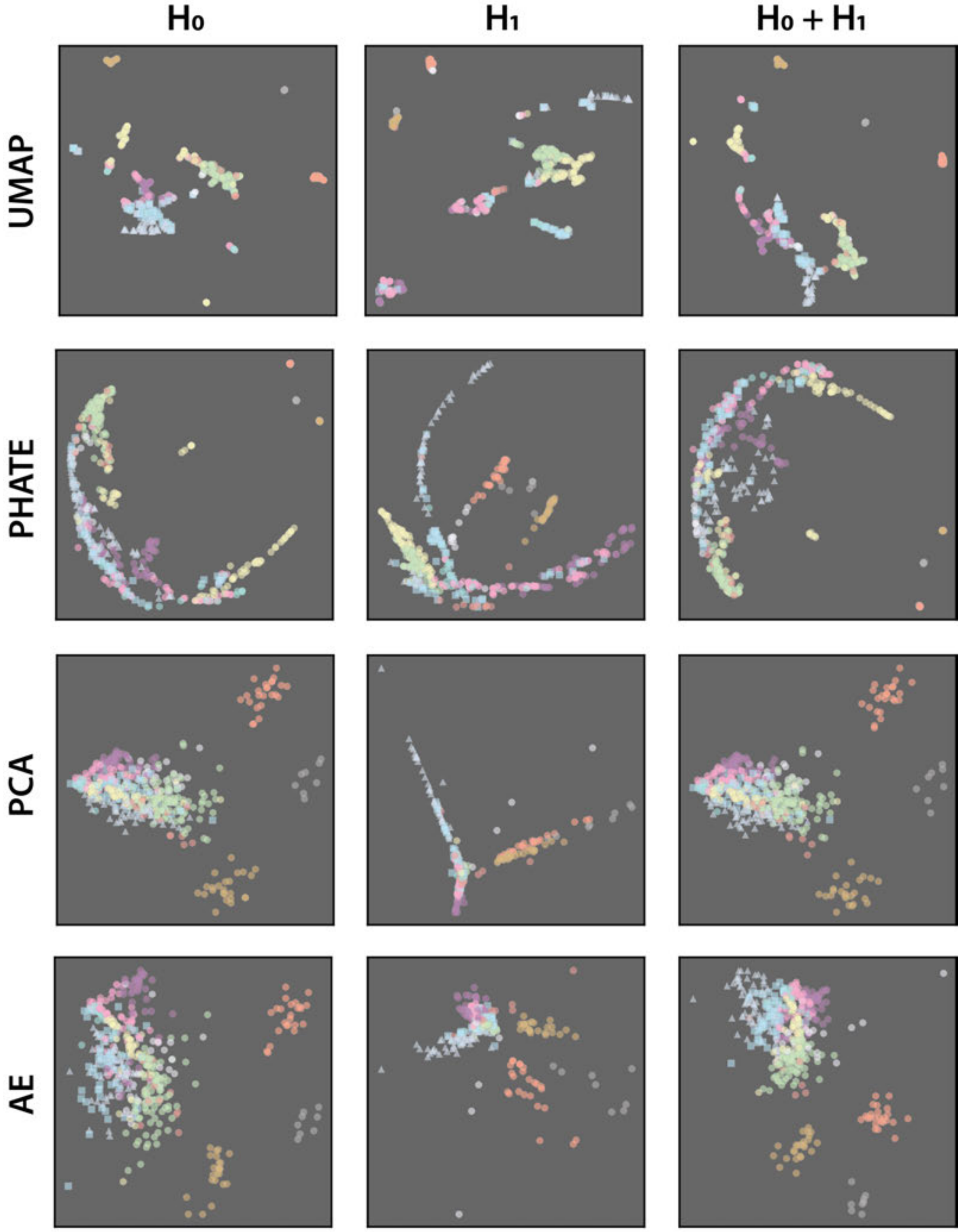

Supplementary Figure 16: **2D embeddings of persistence curves of non-proliferating particle configurations.** Low dimension representation of simulated particle configurations obtained using UMAP, PHATE, and PCA. Embeddings computed using dimension 0, dimension 1, and concatenated persistence curves for simulations with proliferation disabled.

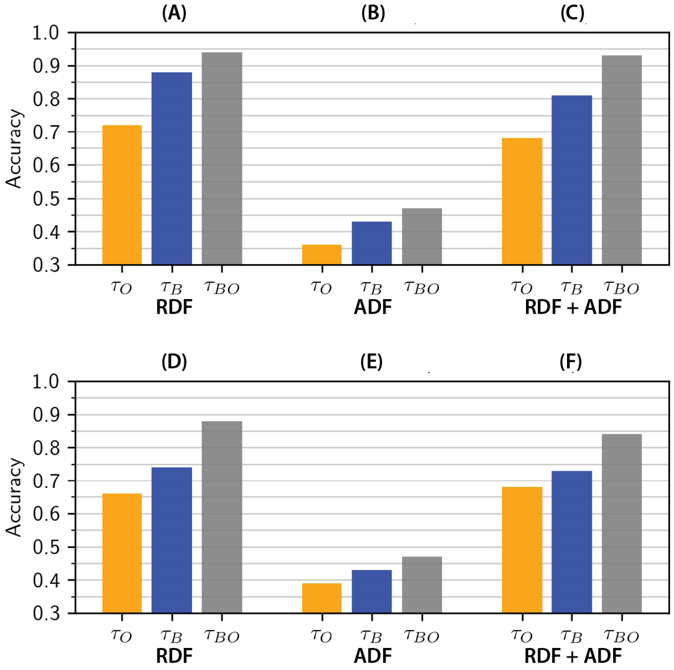

Supplementary Figure 17: **Classification accuracy of radial distribution function (RDF) and angular distribution function (ADF) order parameters.** (A-C) at constant population size. (D-F) at varying population size.

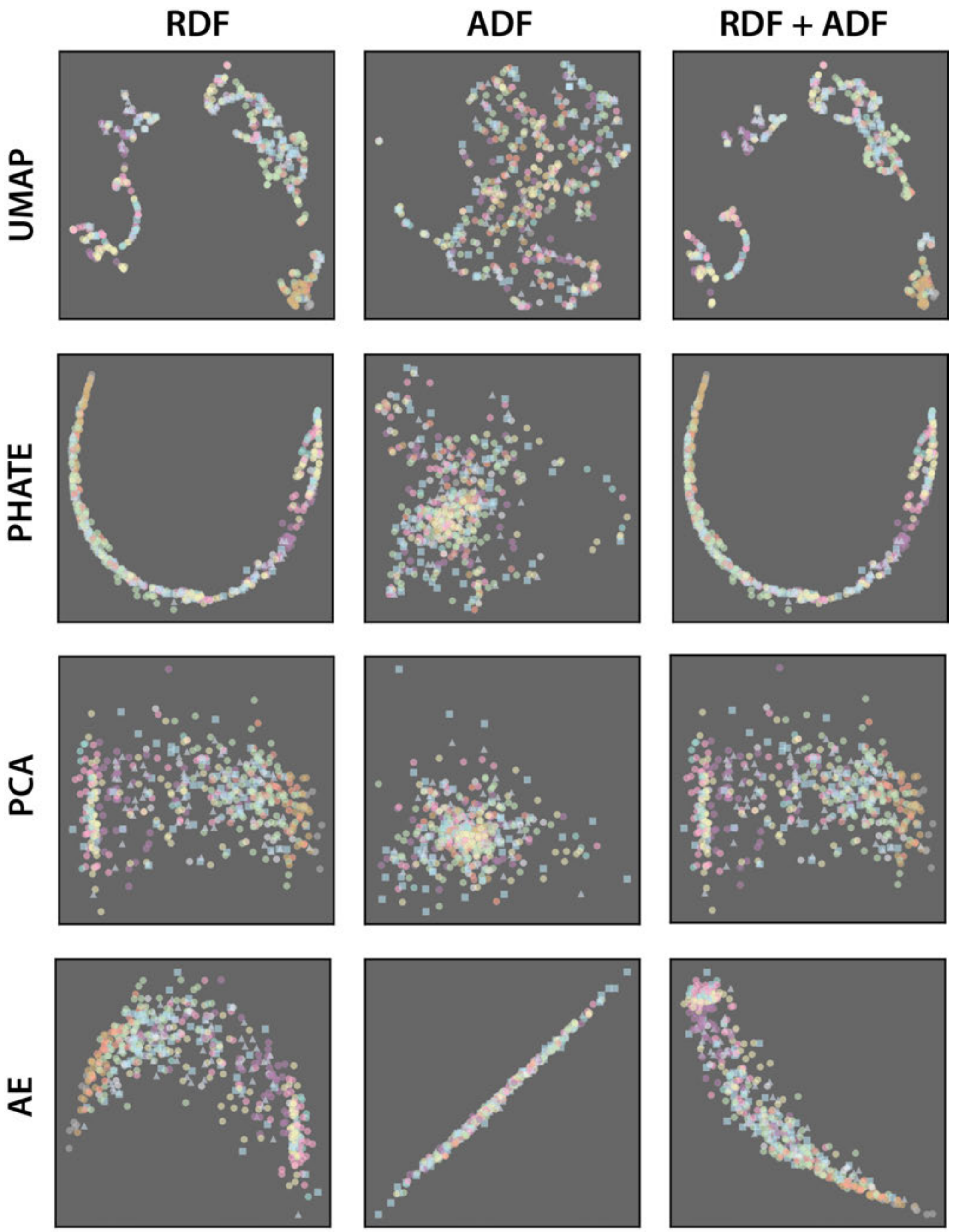

Supplementary Figure 18: **2D embeddings of order parameters of non-proliferating particle configurations.** Low dimension representation of simulated particle configurations obtained using UMAP, PHATE, and PCA. Embeddings computed using radial, angular, and concatenated order parameters for simulations with proliferation disabled.

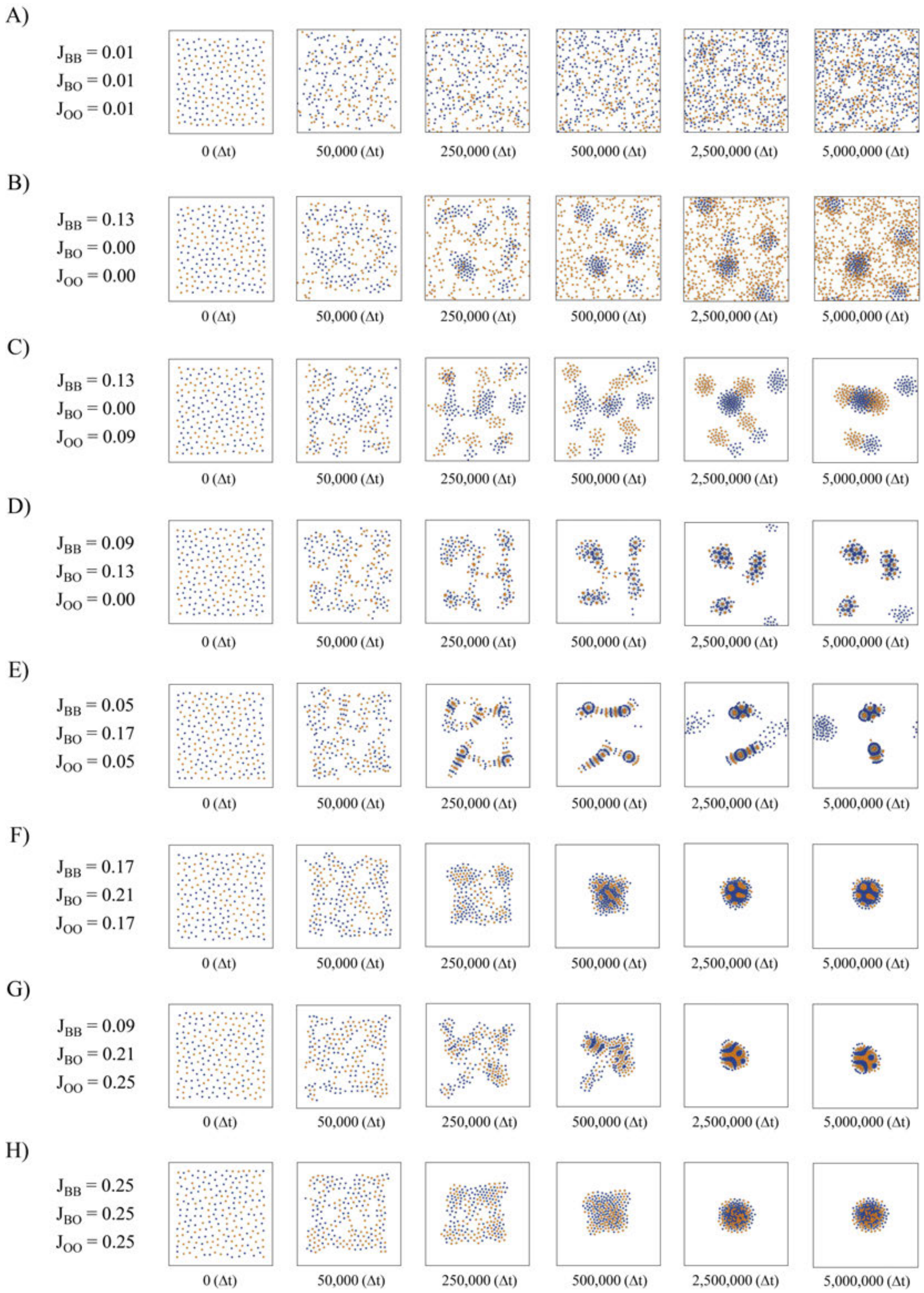

Supplementary Figure 19: **Self-organized pattern formation in time-lapse snapshots (log scale) with proliferation turned on at varying differential adhesion values.** (A) All particles remain individually dispersed in the absence of the adhesion force. (B) Blue particles aggregate into clusters due to high blue-blue adhesion. (C) Complete sorting simulation where both blue and orange particles form separate clusters due to high homotypic adhesion. (D-G) High heterotypic adhesion results in configurations that maximize the interaction between the two cell types, forming hexagonal, striped and spotted patterns. (H) Well-mixed clusters are obtained when homotypic and heterotypic adhesion values are greater than zero and (approximately) equal.

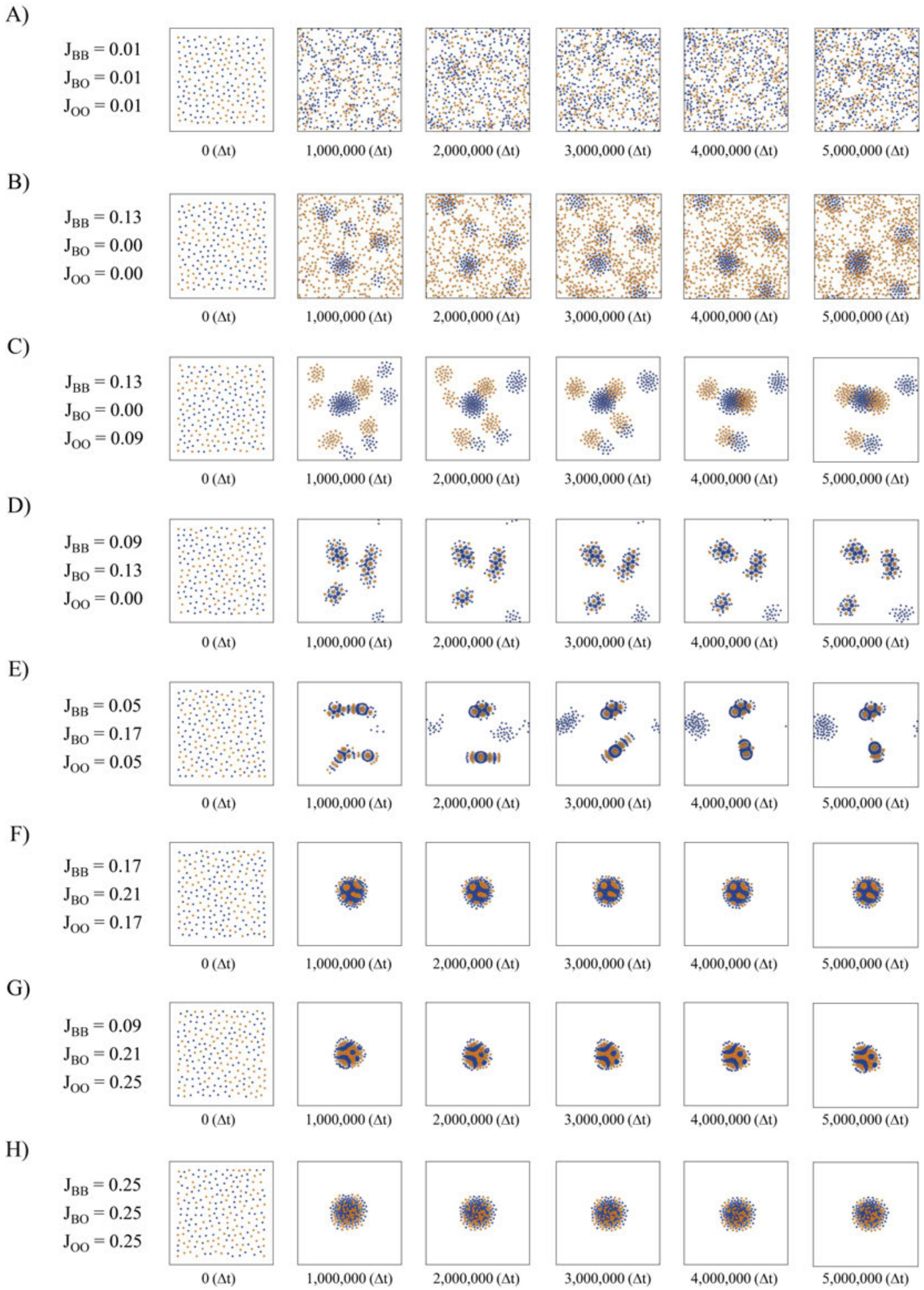

Supplementary Figure 20: **Self-organized pattern formation in time-lapse snapshots (linear scale) with proliferation turned on at varying differential adhesion values.** (A) All particles remain individually dispersed in the absence of the adhesion force. (B) Blue particles aggregate into clusters due to high blue-blue adhesion. (C) Complete sorting simulation where both blue and orange particles form separate clusters due to high homotypic adhesion. (D-G) High heterotypic adhesion results in configurations that maximize the interaction between the two cell types, forming hexagonal, striped and spotted patterns. (H) Well-mixed clusters are obtained when homotypic and heterotypic adhesion values are greater than zero and (approximately) equal.

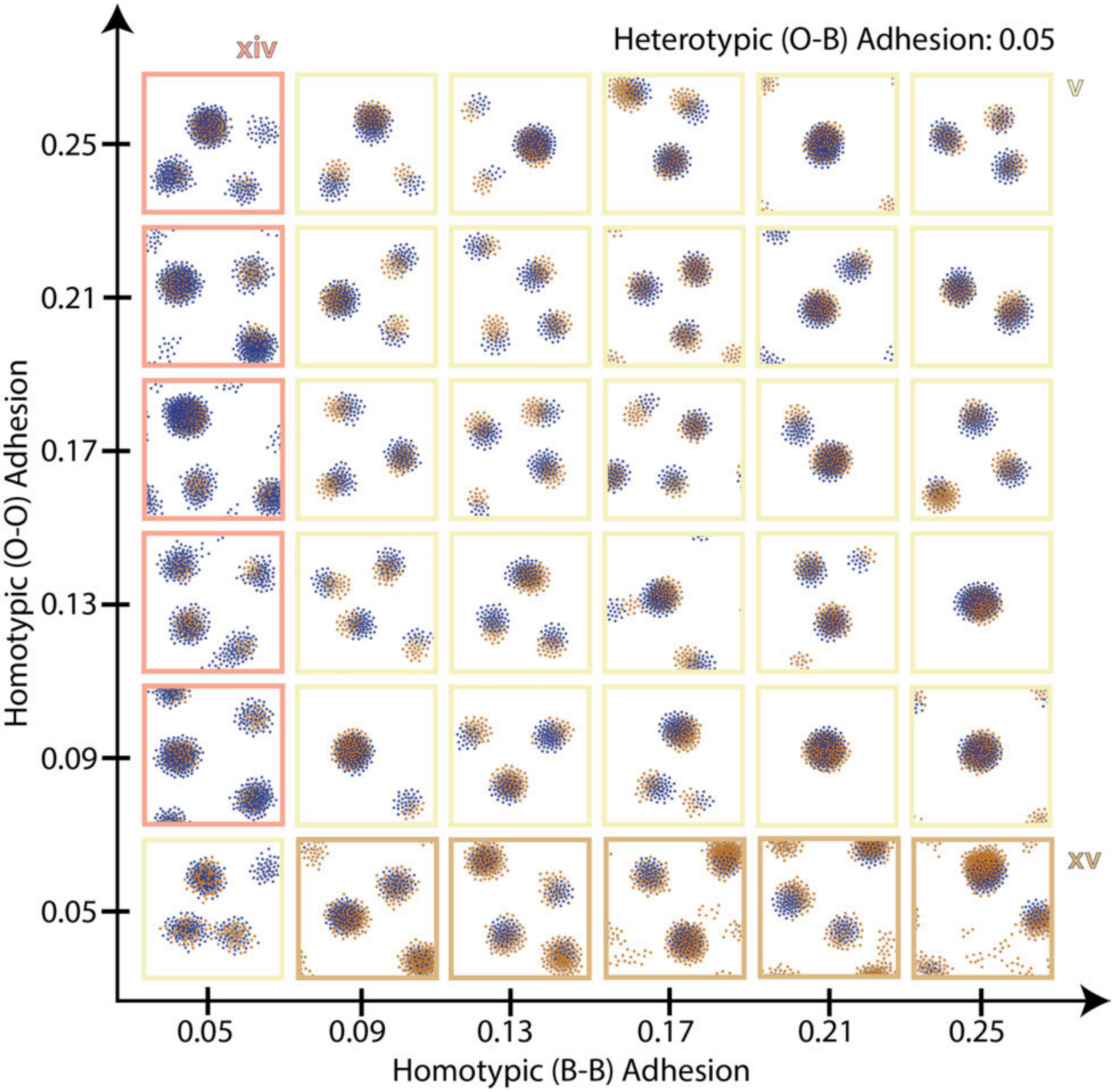

Supplementary Figure 21: **Simulation snapshots of multicellular patterning of a heterogeneous, proliferating population at steady state with $J_{BO} = 0.05$ and varying J_{BB}, J_{OO}.**

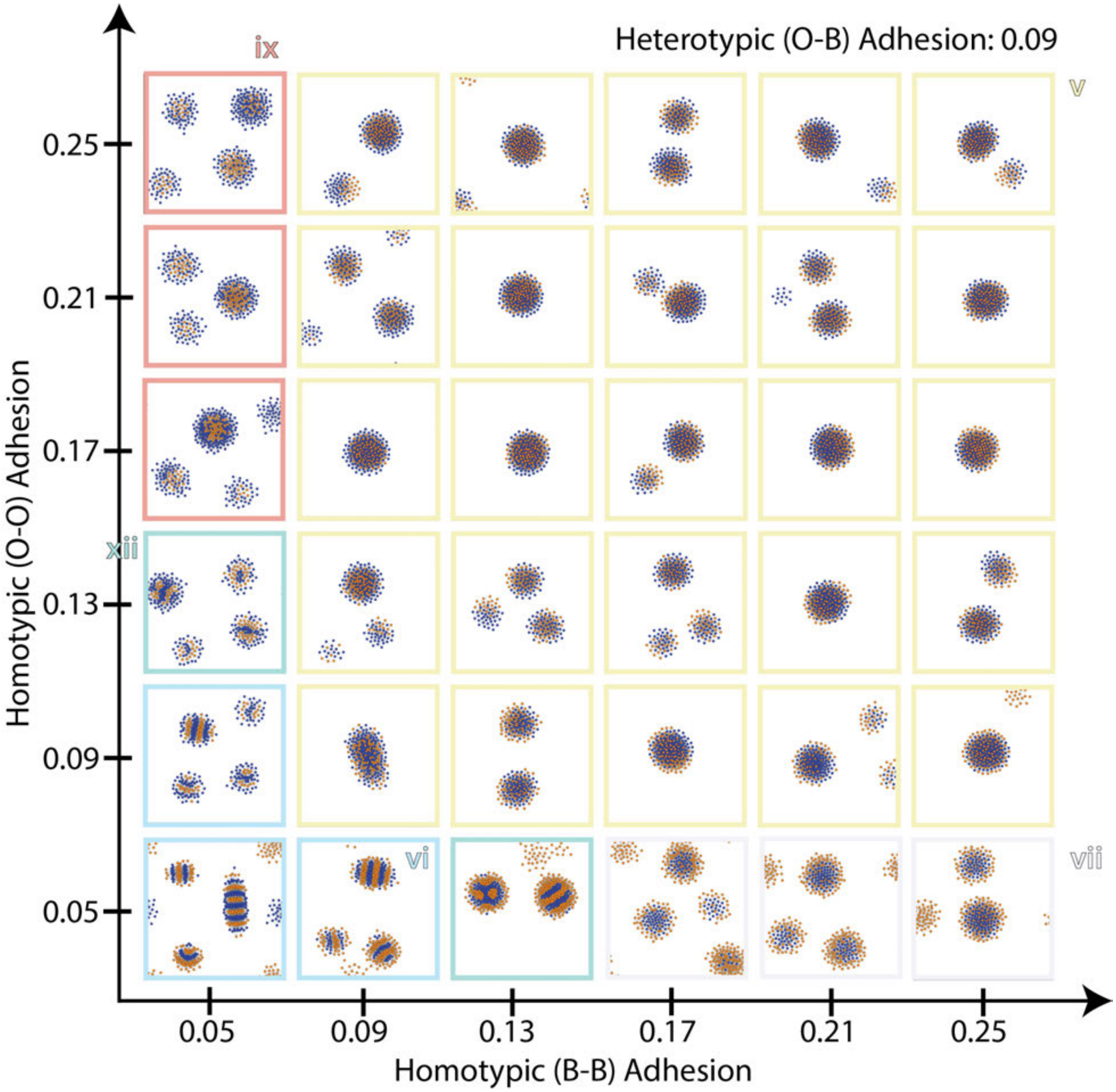

Supplementary Figure 22: **Simulation snapshots of multicellular patterning of a heterogeneous, proliferating population at steady state with $\mathbf{J_{BO} = 0.09}$ and varying $\mathbf{J_{BB}, J_{OO}}$.**

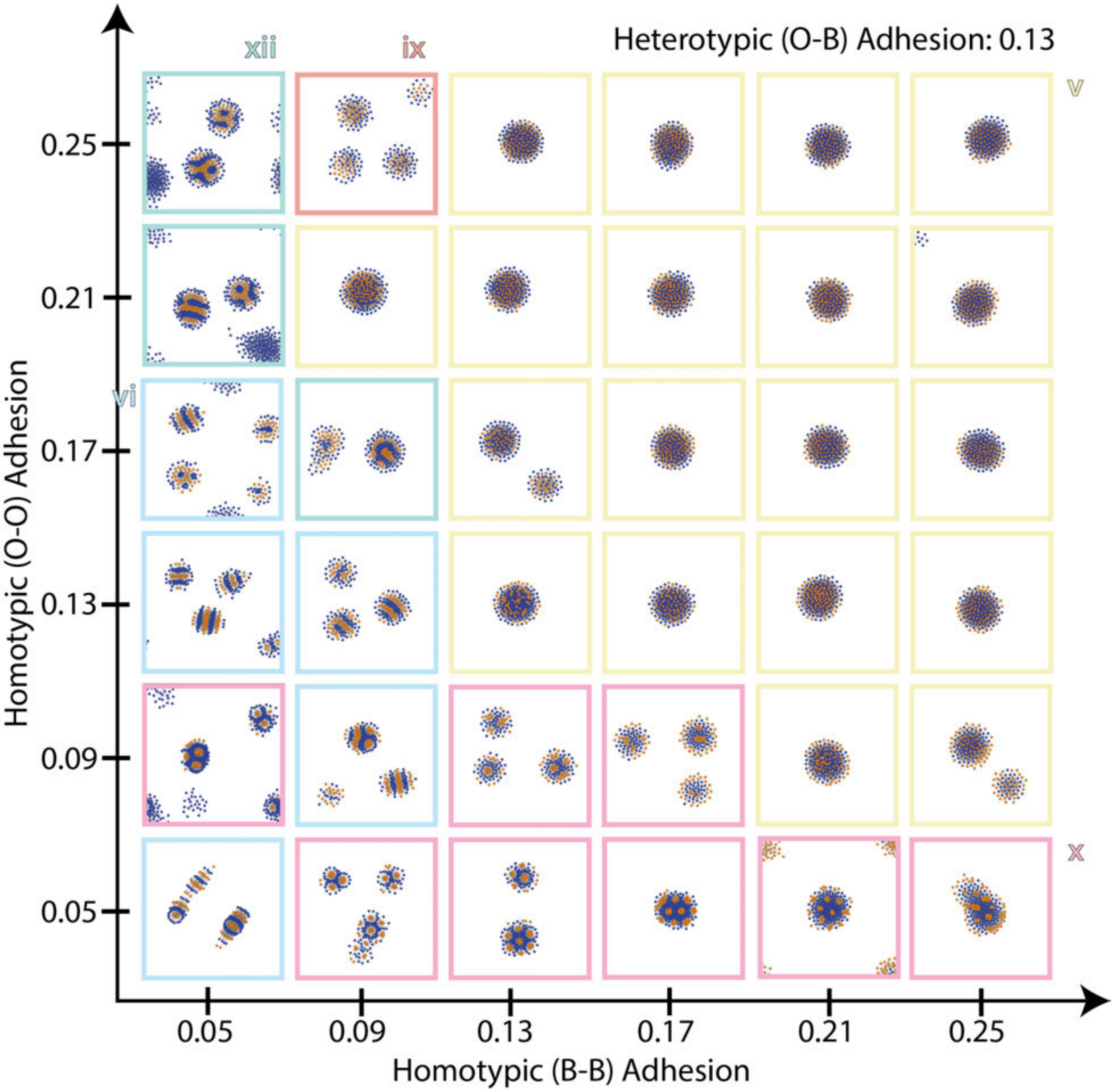

Supplementary Figure 23: **Simulation snapshots of multicellular patterning of a heterogeneous, proliferating population at steady state with $\mathbf{J_{BO} = 0.13}$ and varying $\mathbf{J_{BB}, J_{OO}}$.**

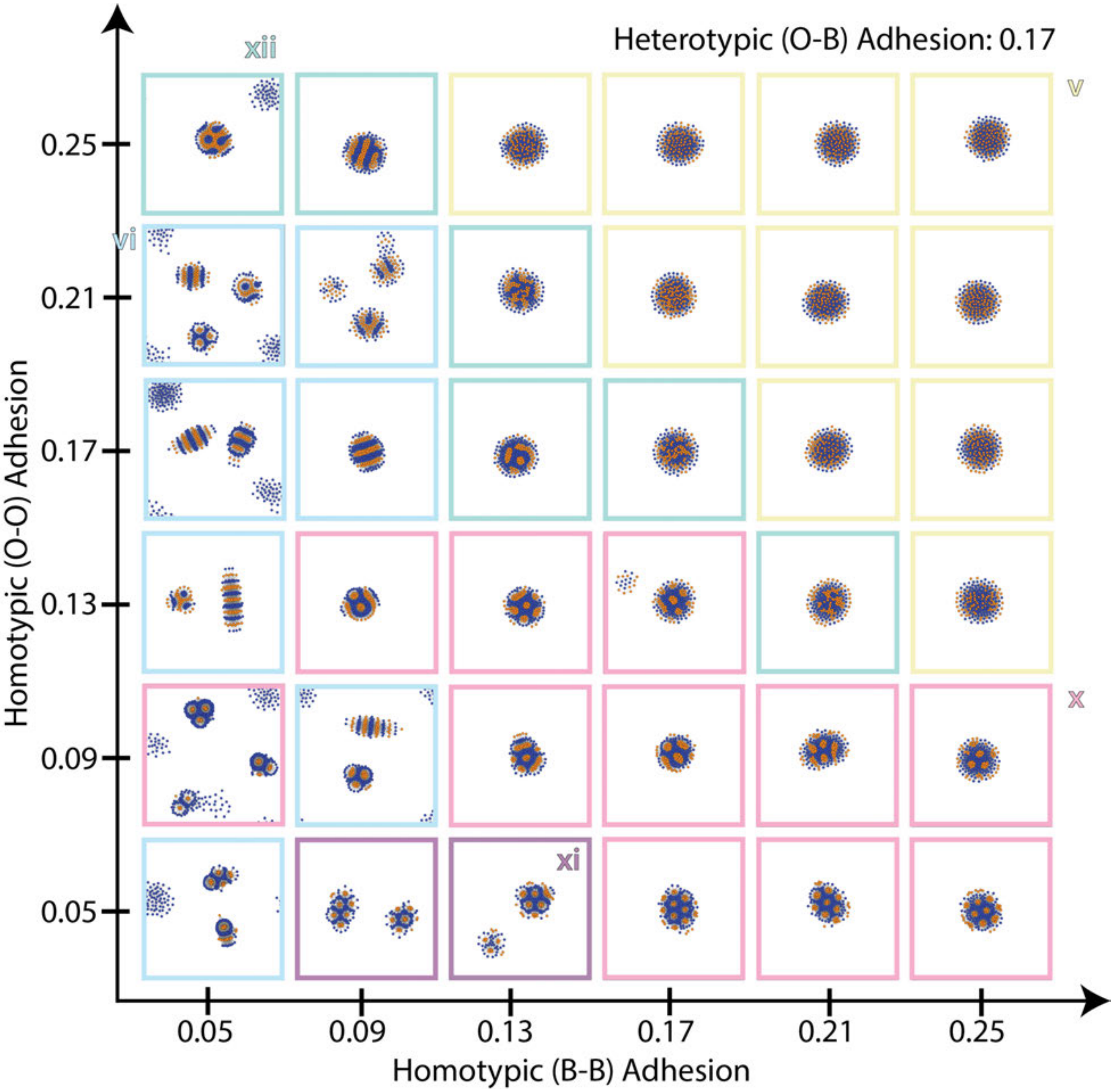

Supplementary Figure 24: **Simulation snapshots of multicellular patterning of a heterogeneous, proliferating population at steady state with $\mathbf{J_{BO} = 0.17}$ and varying $\mathbf{J_{BB}, J_{OO}}$.**

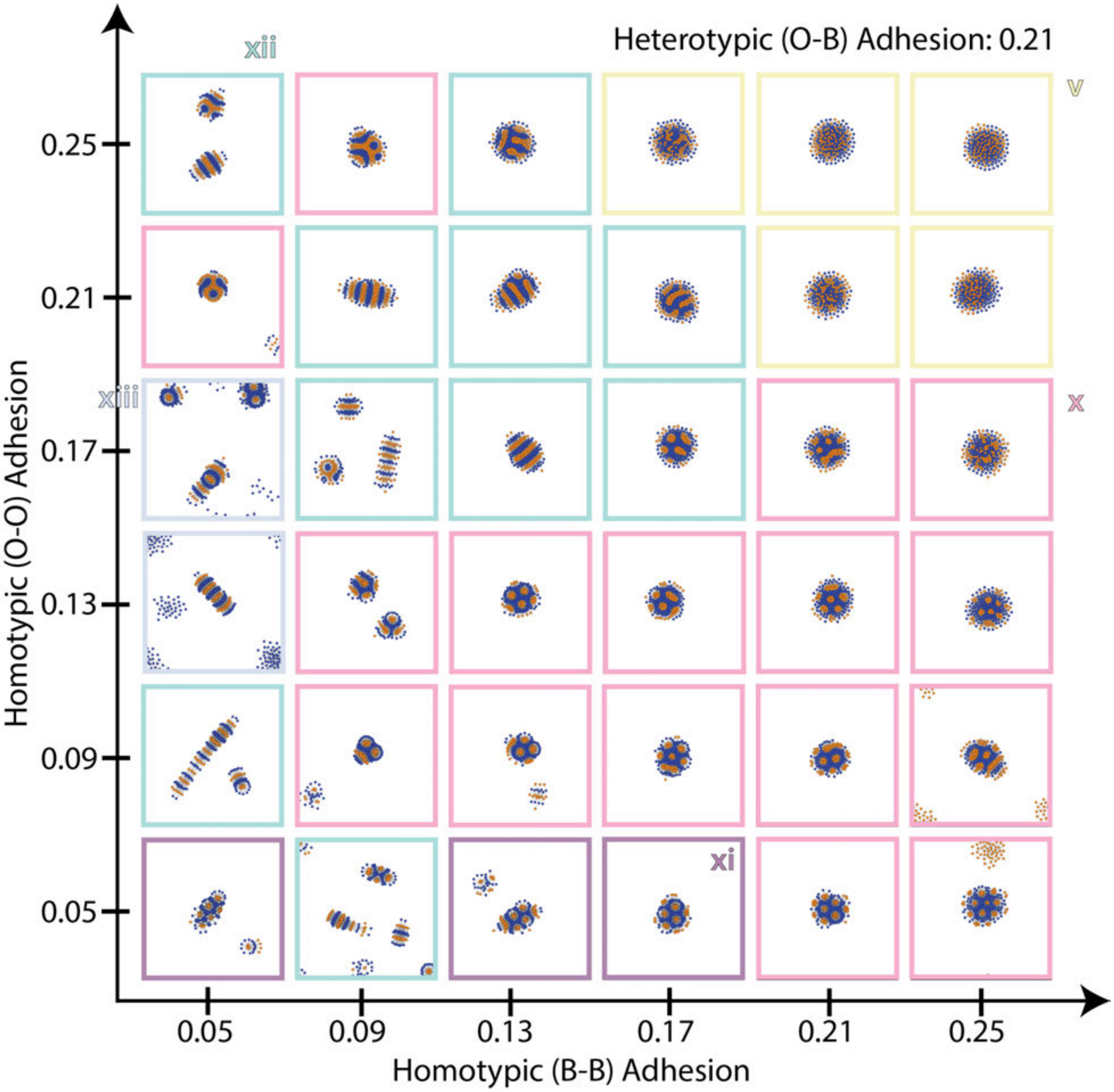

Supplementary Figure 25: **Simulation snapshots of multicellular patterning of a heterogeneous, proliferating population at steady state with $\mathbf{J_{BO} = 0.21}$ and varying $\mathbf{J_{BB}, J_{OO}}$.**

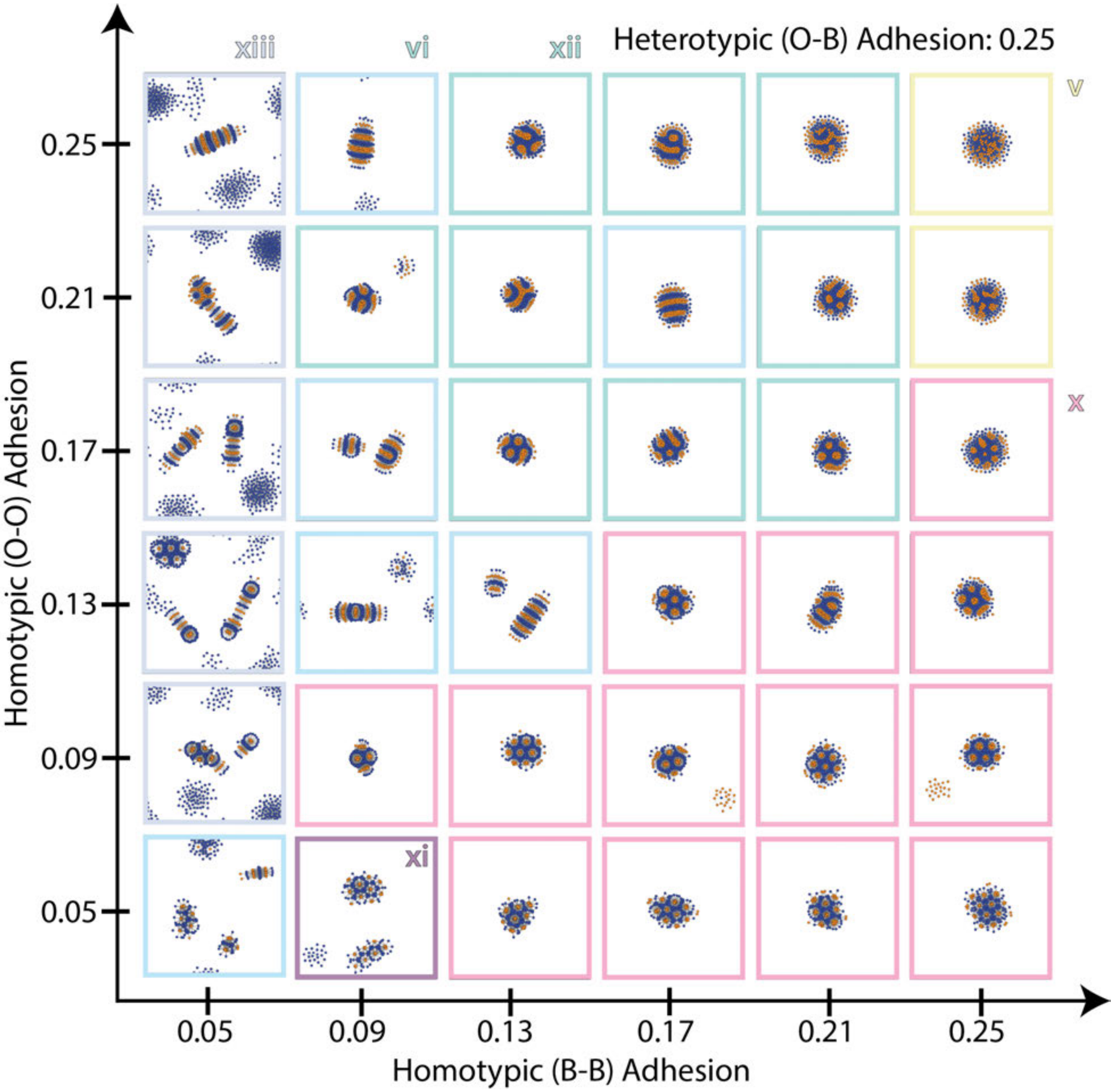

Supplementary Figure 26: **Simulation snapshots of multicellular patterning of a heterogeneous, proliferating population at steady state with $\mathbf{J_{BO} = 0.25}$ and varying $\mathbf{J_{BB}, J_{OO}}$.**

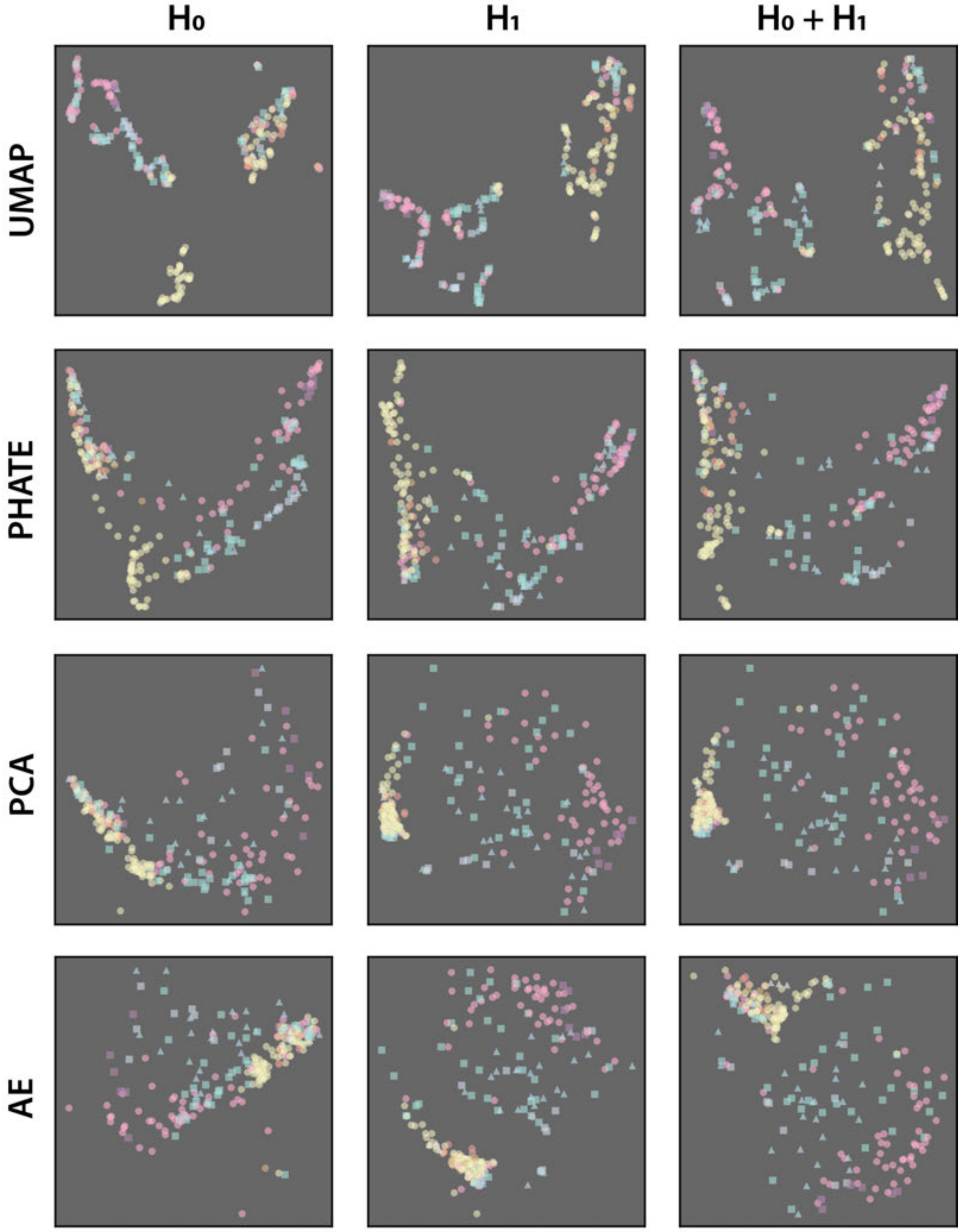

Supplementary Figure 27: **2D embeddings of persistence images of proliferating particle configurations.** Low dimension representation of simulated particle configurations obtained using UMAP, PHATE, and PCA. Embeddings computed using dimension 0, dimension 1, and concatenated persistence images for simulations with proliferation enabled.

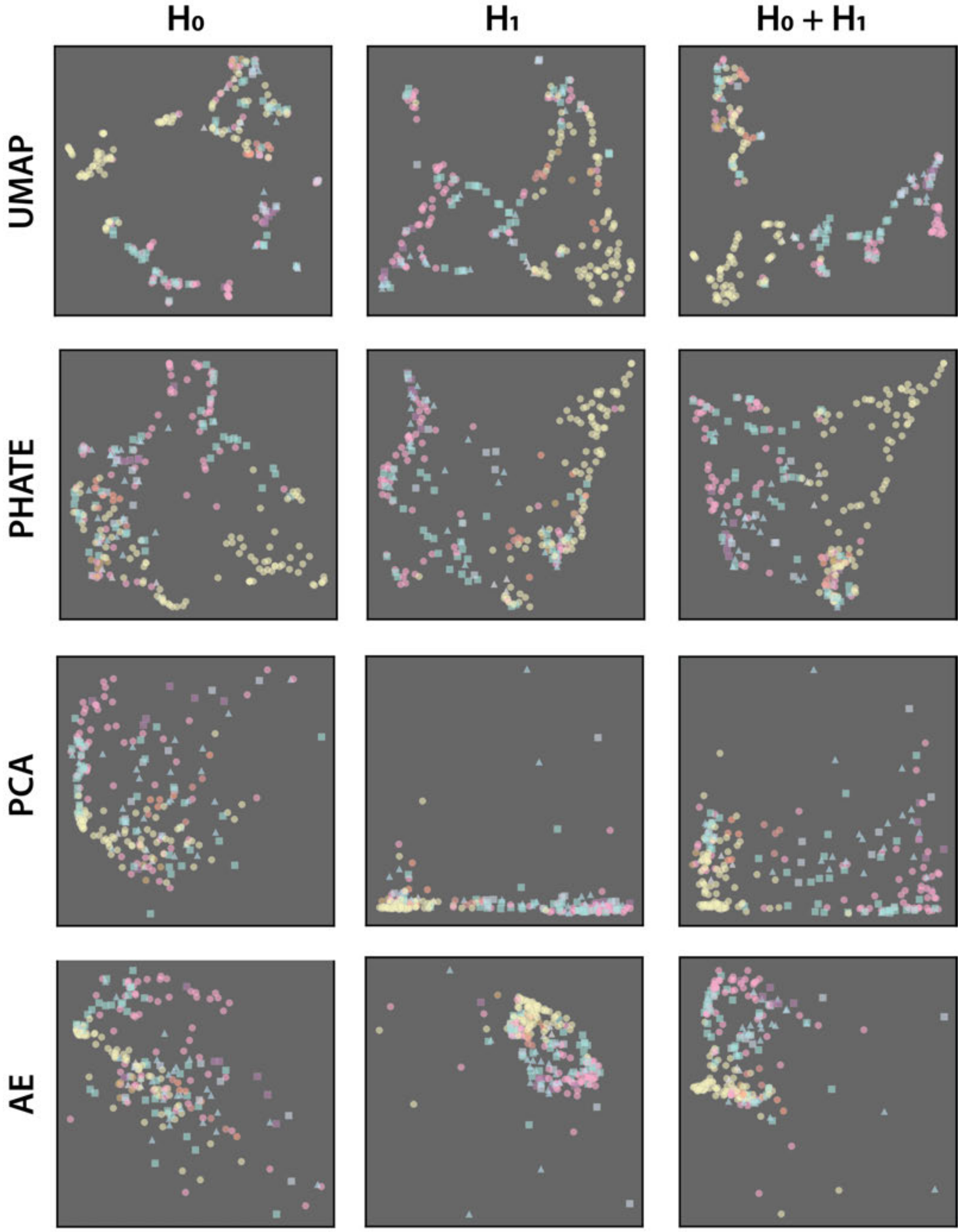

Supplementary Figure 28: **2D embeddings of persistence curves of proliferating particle configurations.** Low dimension representation of simulated particle configurations obtained using UMAP, PHATE, and PCA. Embeddings computed using dimension 0, dimension 1, and concatenated persistence curves for simulations with proliferation enabled.

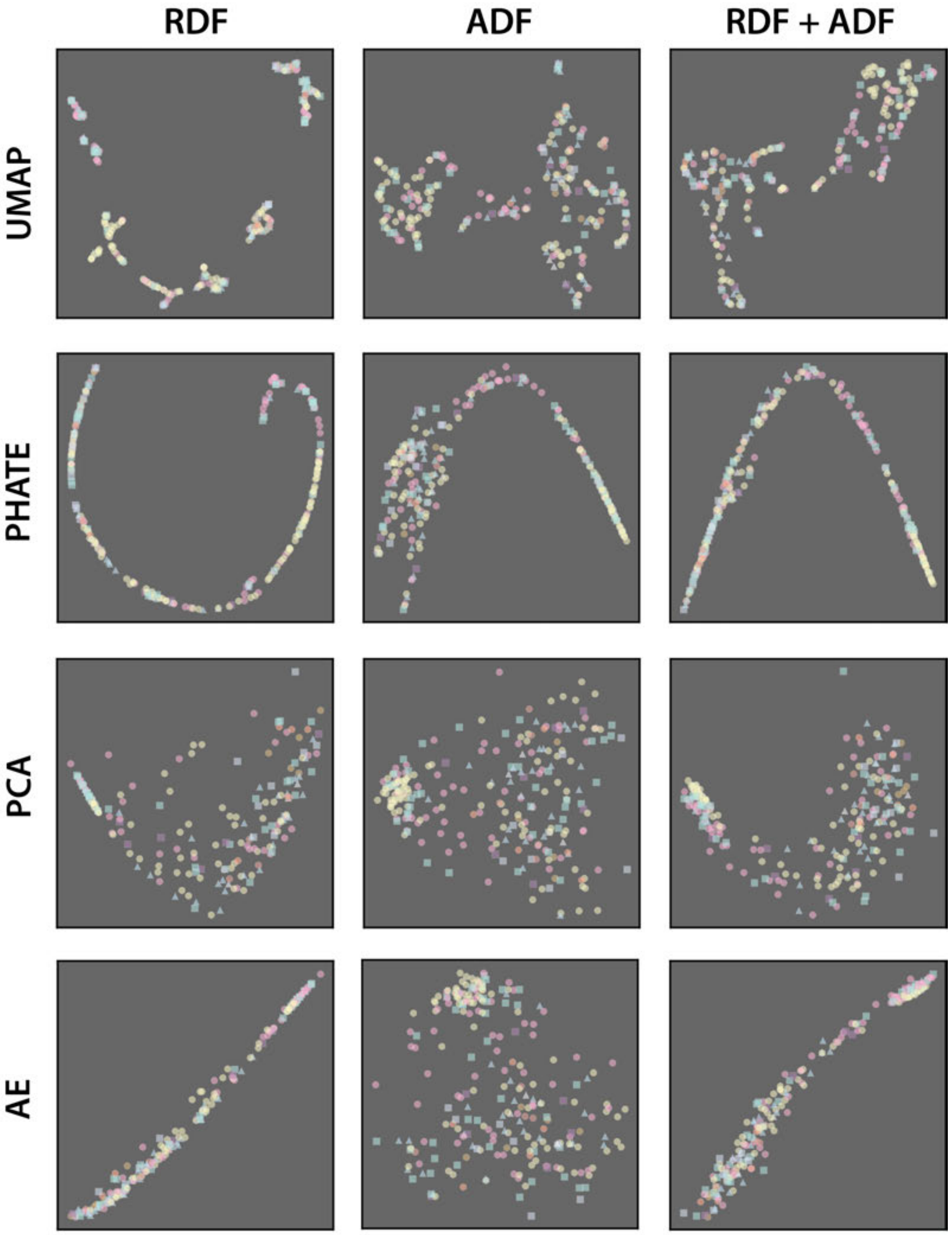

Supplementary Figure 29: **2D embeddings of order parameters of proliferating particle configurations.** Low dimension representation of simulated particle configurations obtained using UMAP, PHATE, and PCA. Embeddings computed using radial, angular, and concatenated order parameters for simulations with proliferation enabled.

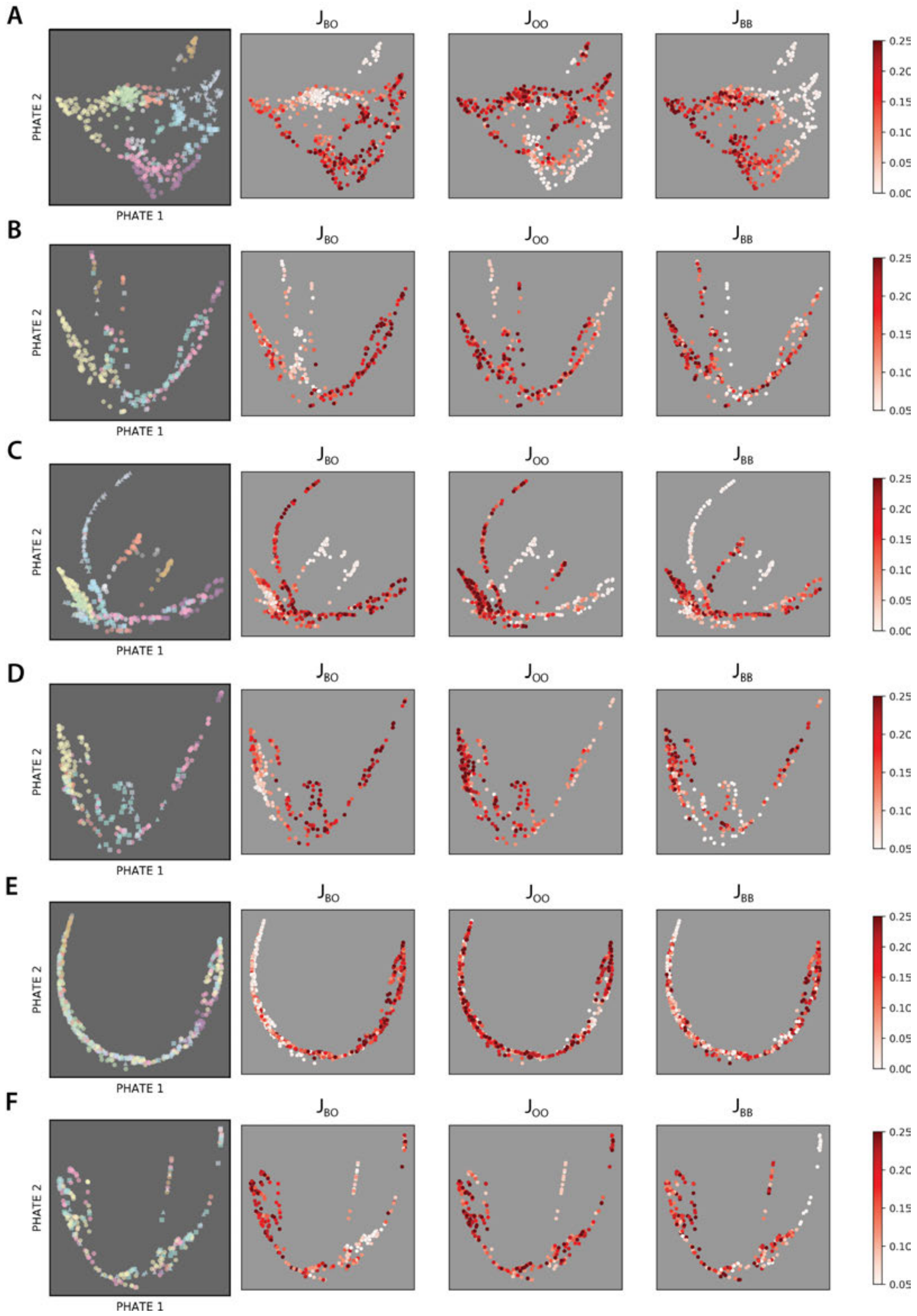

Supplementary Figure 30: **Low-dimensional PHATE representations of simulations and corresponding adhesion values.** PHATE plots constructed using persistence images (A, B), persistence curves (C, D), and order parameters (E, F) colored by ground truth classification (left) and adhesion parameter values. Persistence images and curves are generated using dimension 1 topological features and order parameters include both radial and angular distributions. Panels A, C, and E correspond to simulations without proliferation. Panels B, D, and F correspond to simulations with proliferation enabled.

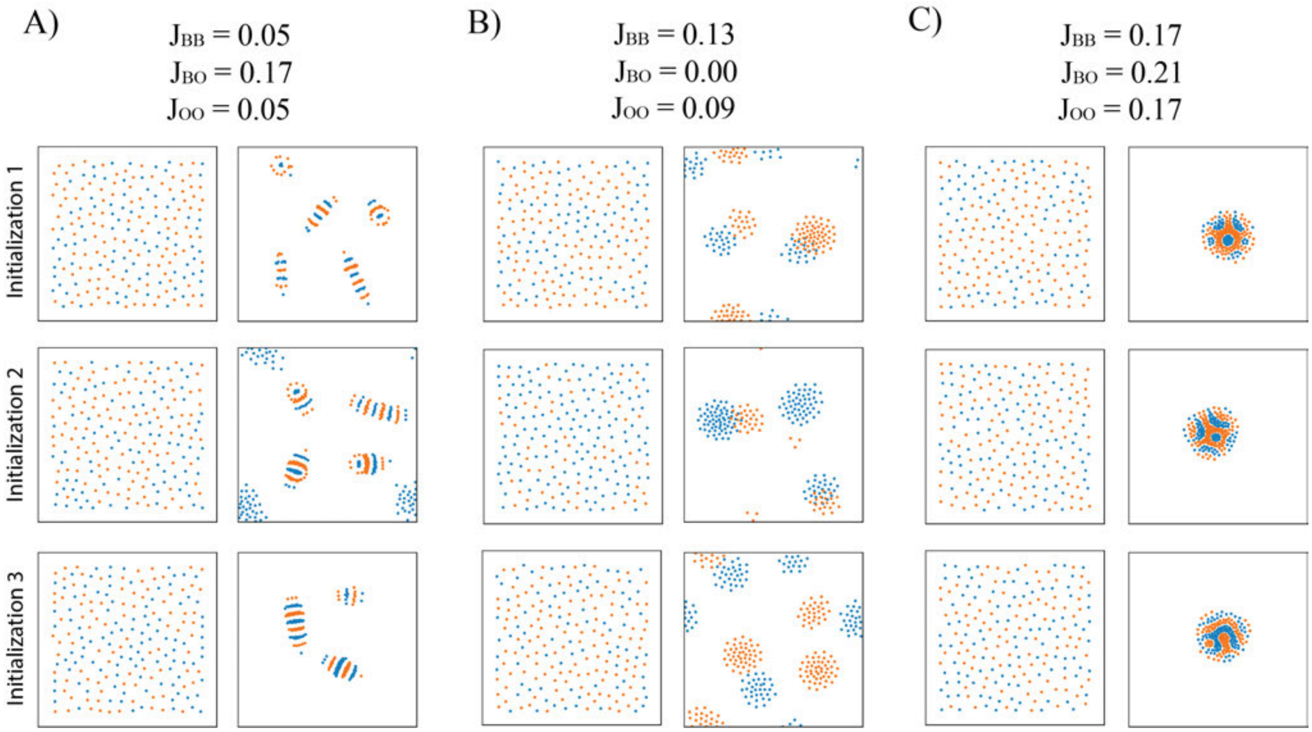

Supplementary Figure 31: **Reproducibility of steady-state configurations at varying initial conditions.** (A) High heterotypic adhesion results in the formation of a stripe pattern where blue and orange particles maximize their interaction with each other. (B) Complete sorting simulation where both blue and orange particles form separate clusters due to high homotypic adhesion. (C) Clustering of blue and orange particles at intermediate homotypic and high heterotypic adhesions.

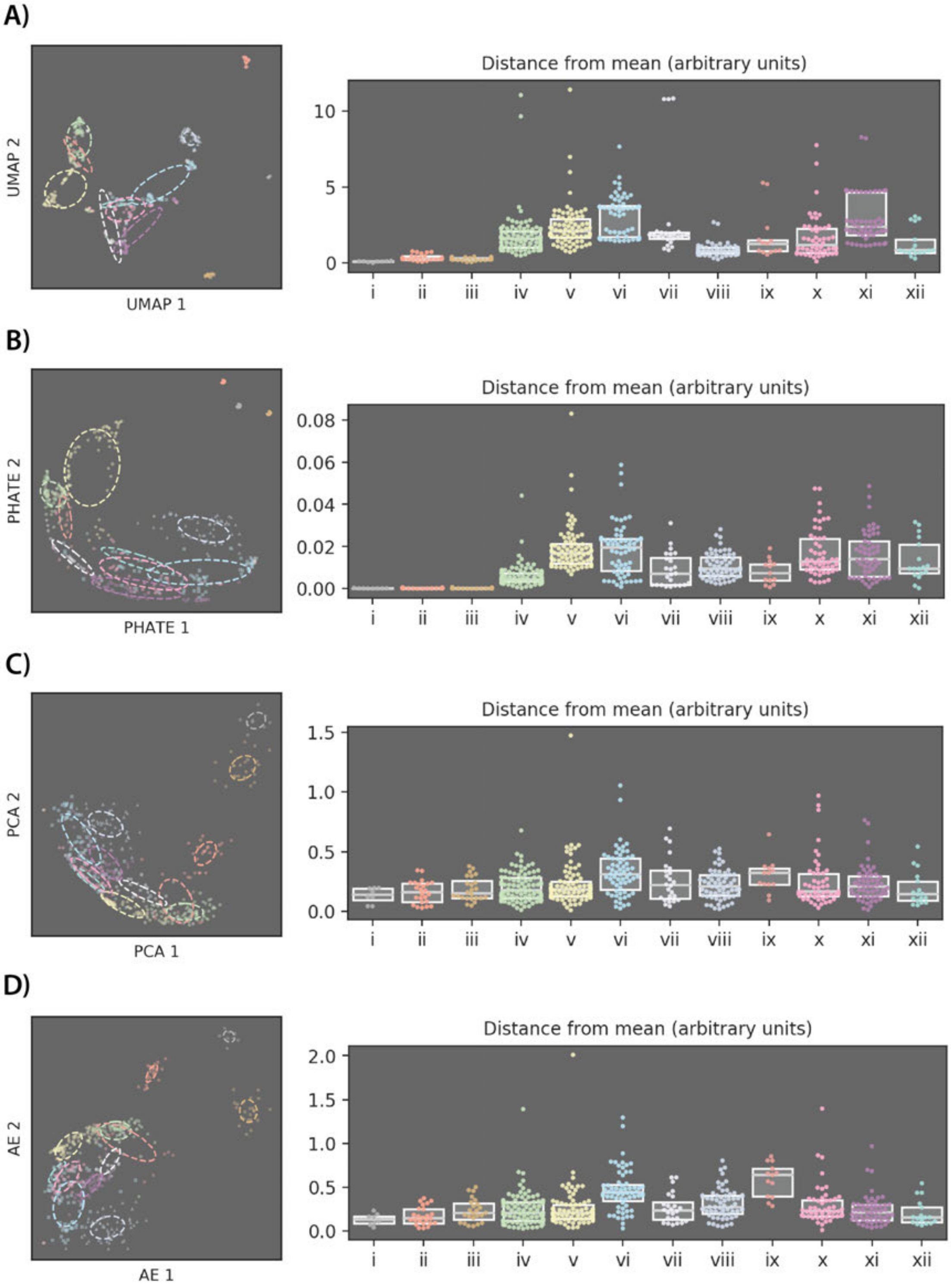

Supplementary Figure 32: **GMM fit to low-dimensional embeddings of connected components in persistent images at constant population size.** Dimension reduced topology of connected components extracted using dimension 0 homology of cell configurations at constant population size. Colored dashed ellipses correspond to unit standard deviation in the Gaussian fit for each cell configuration. Beeswarm and boxplot of distance from mean (cluster center) for each configuration using (A) UMAP, (B) PHATE, (C) PCA, and (D) AE embeddings. The boxplot central line denotes the median, the bottom and top of the box correspond to the 25th and 75th percentiles, respectively.

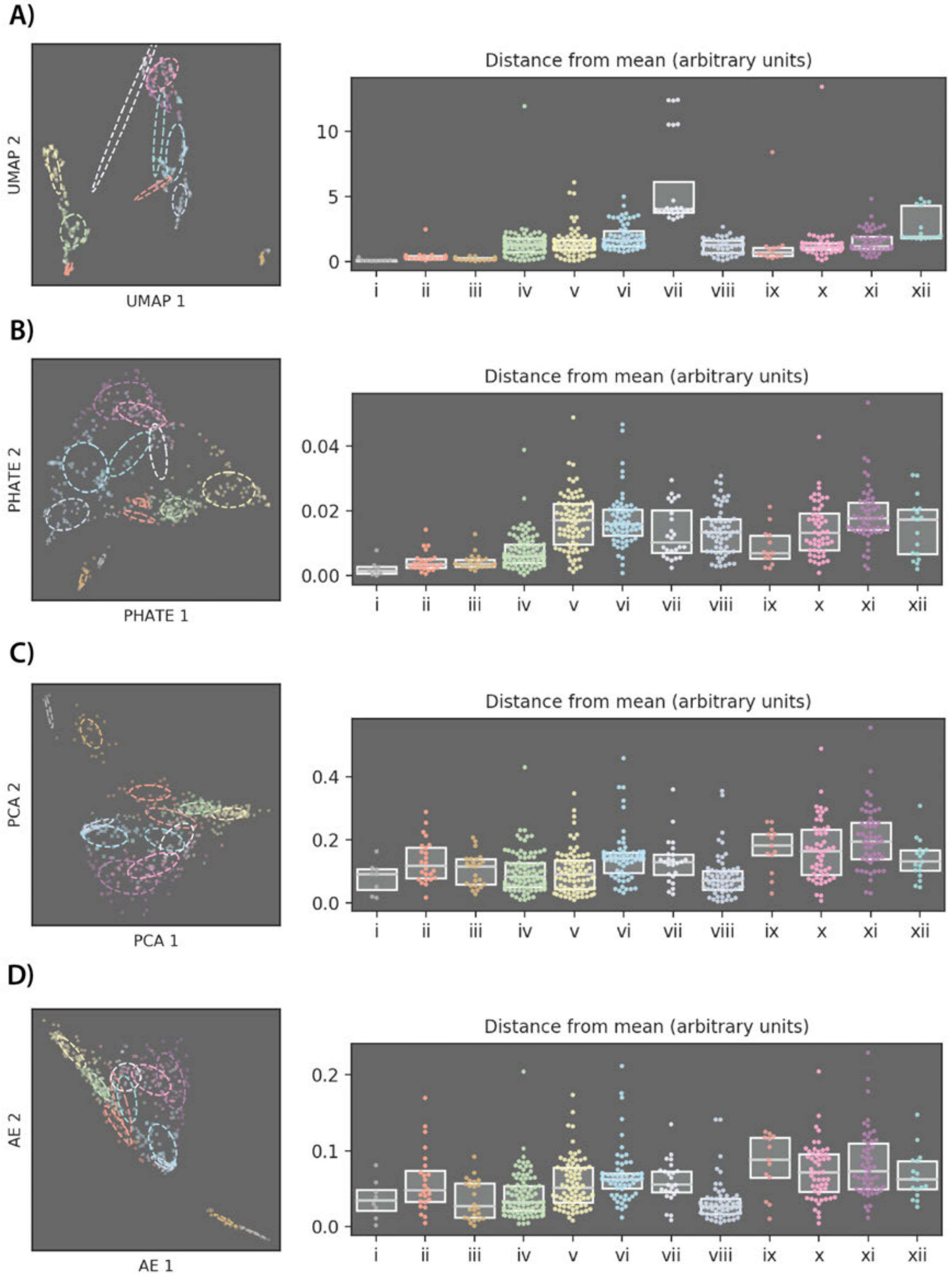

Supplementary Figure 33: **GMM fit to low-dimensional embeddings of topological loops in persistent images at constant population size.** Dimension reduced topology of loops extracted using dimension 1 homology of cell configurations at constant population size. Colored dashed ellipses correspond to unit standard deviation in the Gaussian fit for each cell configuration. Beeswarm and boxplot of distance from mean (cluster center) for each configuration using (A) UMAP, (B) PHATE, (C) PCA, and (D) AE embeddings. The boxplot central line denotes the median, the bottom and top of the box correspond to the 25th and 75th percentiles, respectively.

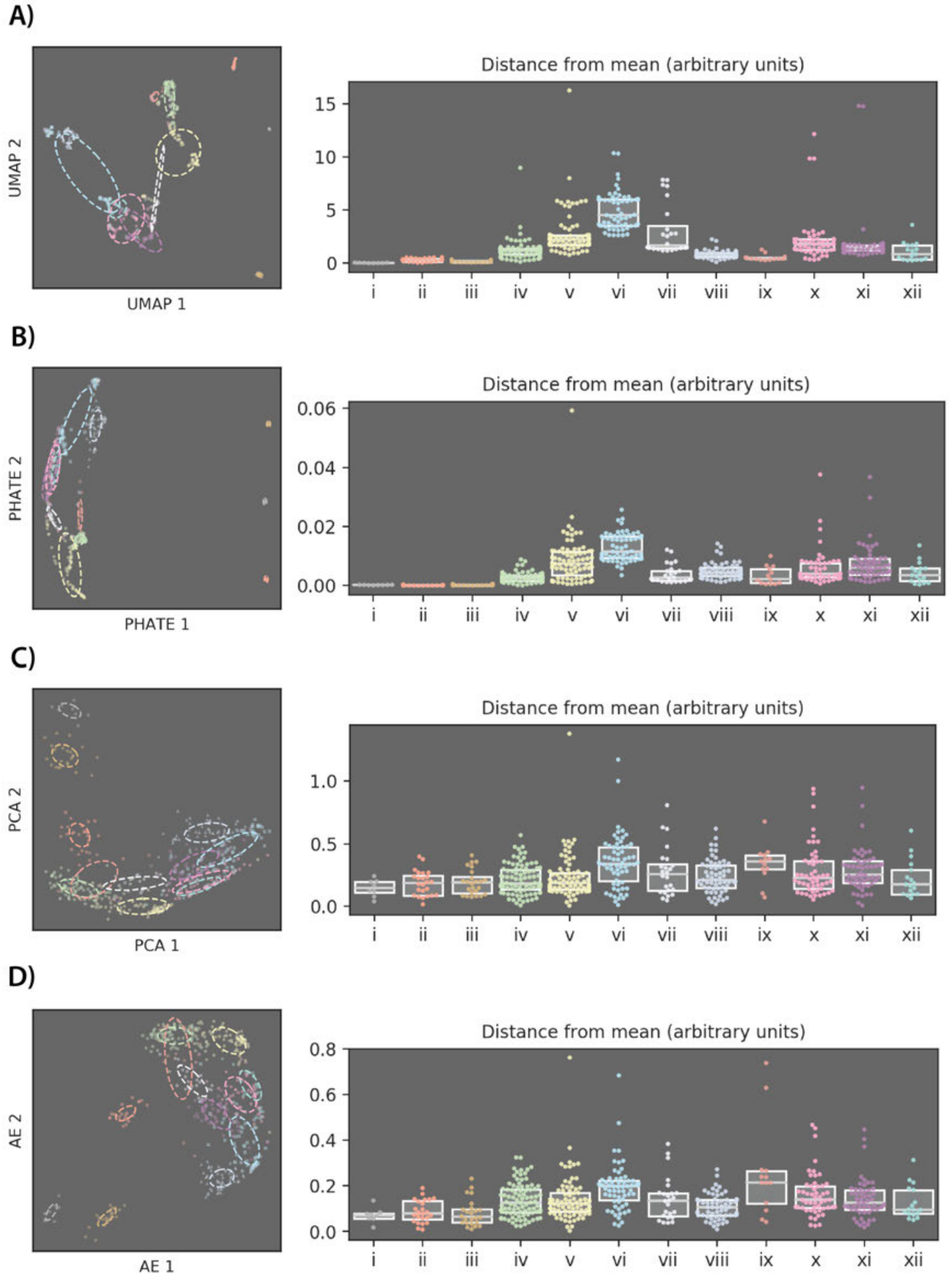

Supplementary Figure 34: **GMM fit to low-dimensional embeddings of concatenated topological features in persistent images at constant population size.** Dimension reduced topology of connected components and loops extracted using concatenated dimension 0 and dimension 1 homology of cell configurations at constant population size. Colored dashed ellipses correspond to unit standard deviation in the Gaussian fit for each cell configuration. Beeswarm and boxplot of distance from mean (cluster center) for each configuration using (A) UMAP, (B) PHATE, (C) PCA, and (D) AE embeddings. The boxplot central line denotes the median, the bottom and top of the box correspond to the 25th and 75th percentiles, respectively.

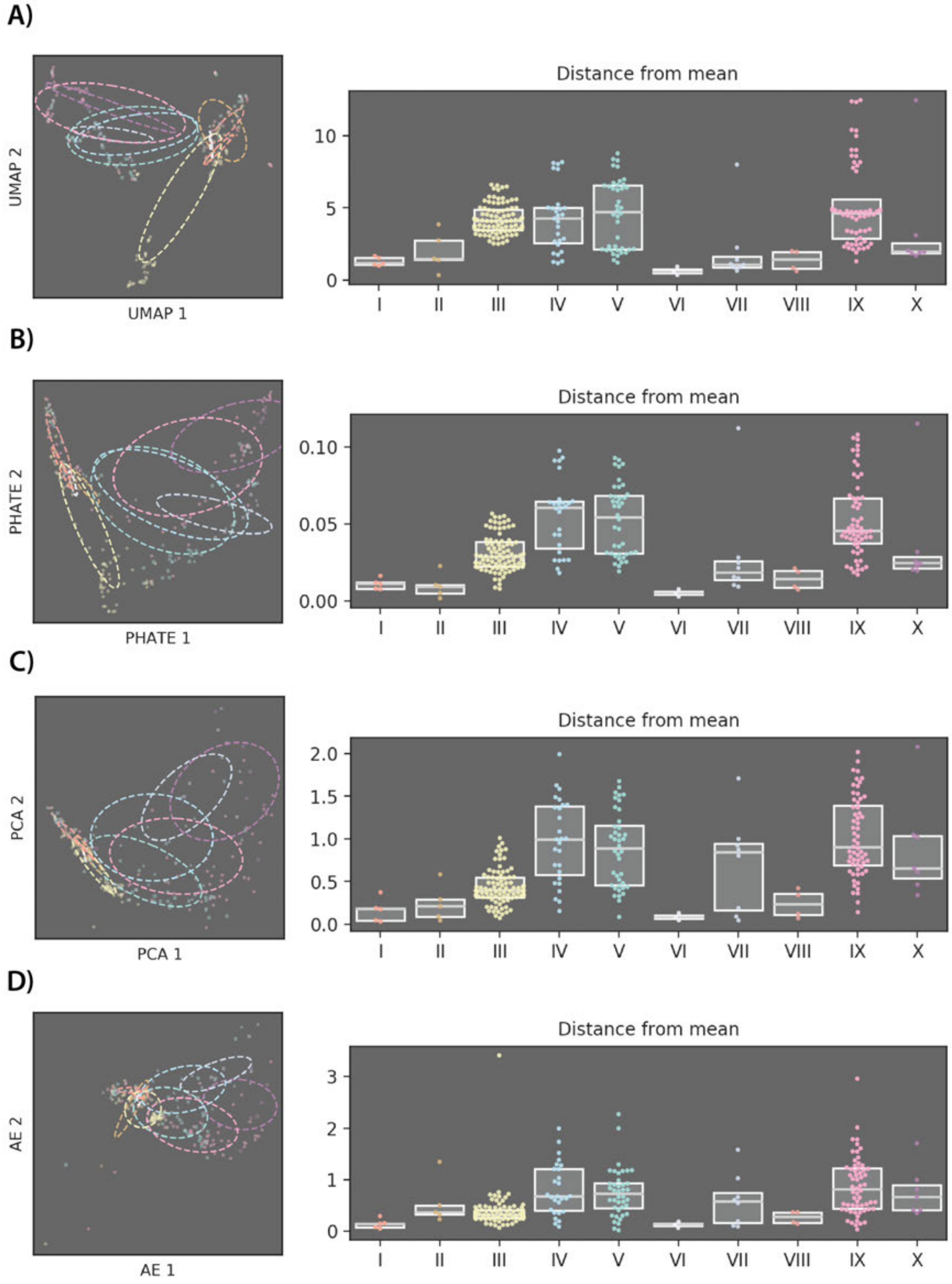

Supplementary Figure 35: **GMM fit to low-dimensional embeddings of connected components in persistent images at varying population size.** Dimension reduced topology of connected components extracted using dimension 0 homology of cell configurations at varying population size. Colored dashed ellipses correspond to unit standard deviation in the Gaussian fit for each cell configuration. Beeswarm and boxplot of distance from mean (cluster center) for each configuration using (A) UMAP, (B) PHATE, (C) PCA, and (D) AE embeddings. The boxplot central line denotes the median, the bottom and top of the box correspond to the 25th and 75th percentiles, respectively.

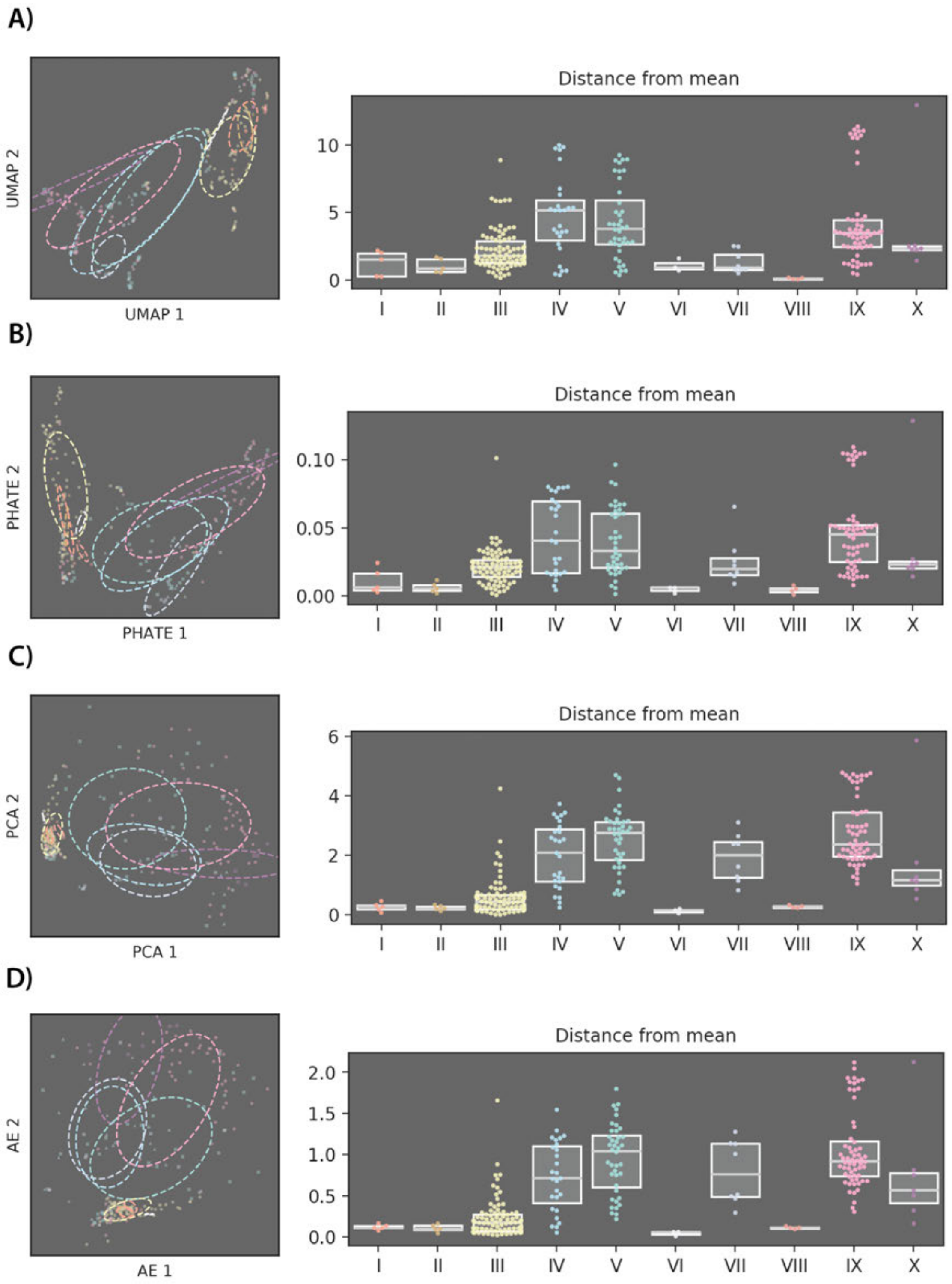

Supplementary Figure 36: **GMM fit to low-dimensional embeddings of topological loops in persistent images at varying population size.** Dimension reduced topology of loops extracted using dimension 1 homology of cell configurations at varying population size. Colored dashed ellipses correspond to unit standard deviation in the Gaussian fit for each cell configuration. Beeswarm and boxplot of distance from mean (cluster center) for each configuration using (A) UMAP, (B) PHATE, (C) PCA, and (D) AE embeddings. The boxplot central line denotes the median, the bottom and top of the box correspond to the 25th and 75th percentiles, respectively.

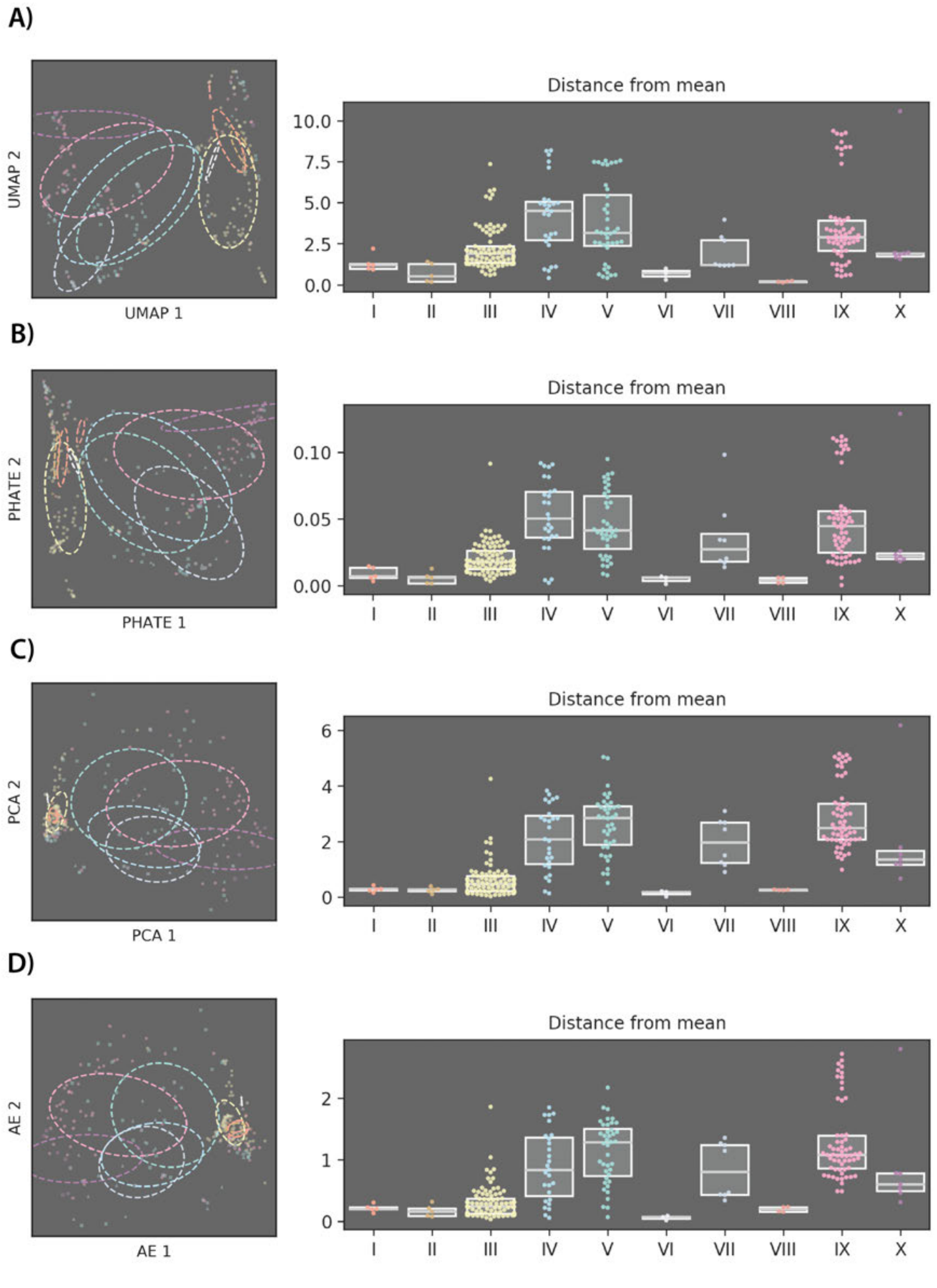

Supplementary Figure 37: **GMM fit to low-dimensional embeddings of concatenated topological features in persistent images at varying population size.** Dimension reduced topology of connected components and loops extracted using concatenated dimension 0 and dimension 1 homology of cell configurations at varying population size. Colored dashed ellipses correspond to unit standard deviation in the Gaussian fit for each cell configuration. Beeswarm and boxplot of distance from mean (cluster center) for each configuration using (A) UMAP, (B) PHATE, (C) PCA, and (D) AE embeddings. The boxplot central line denotes the median, the bottom and top of the box correspond to the 25th and 75th percentiles, respectively.

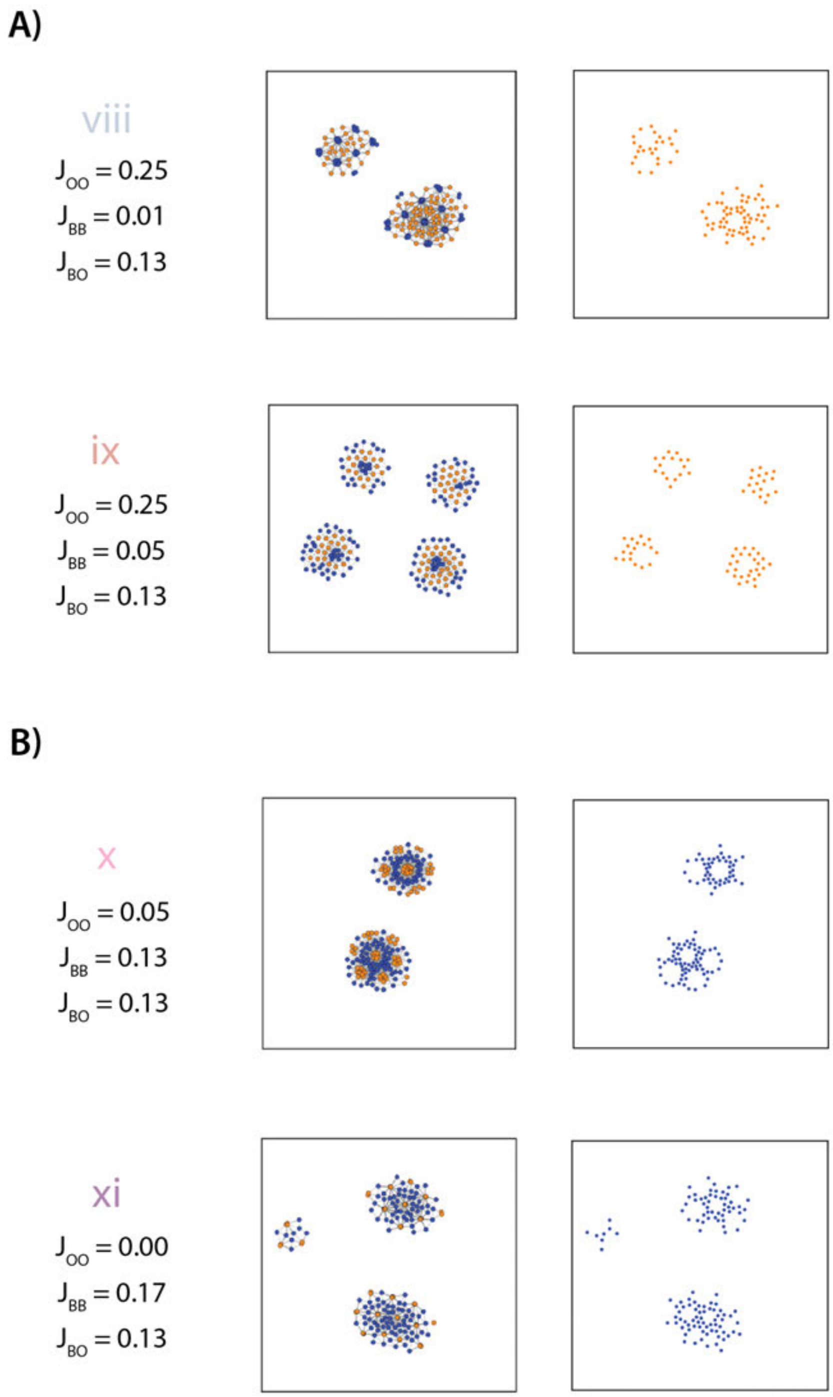

Supplementary Figure 38: **Examples of misclassified cell configurations due to similarity of single-cell topological loops.** (A) Simulations are misclassified when only orange cell positions are considered. (B) Simulations are misclassified when only blue cell positions are considered. Note that these simulations are not misclassified in dimension 0 topology, due to differences in cell density.

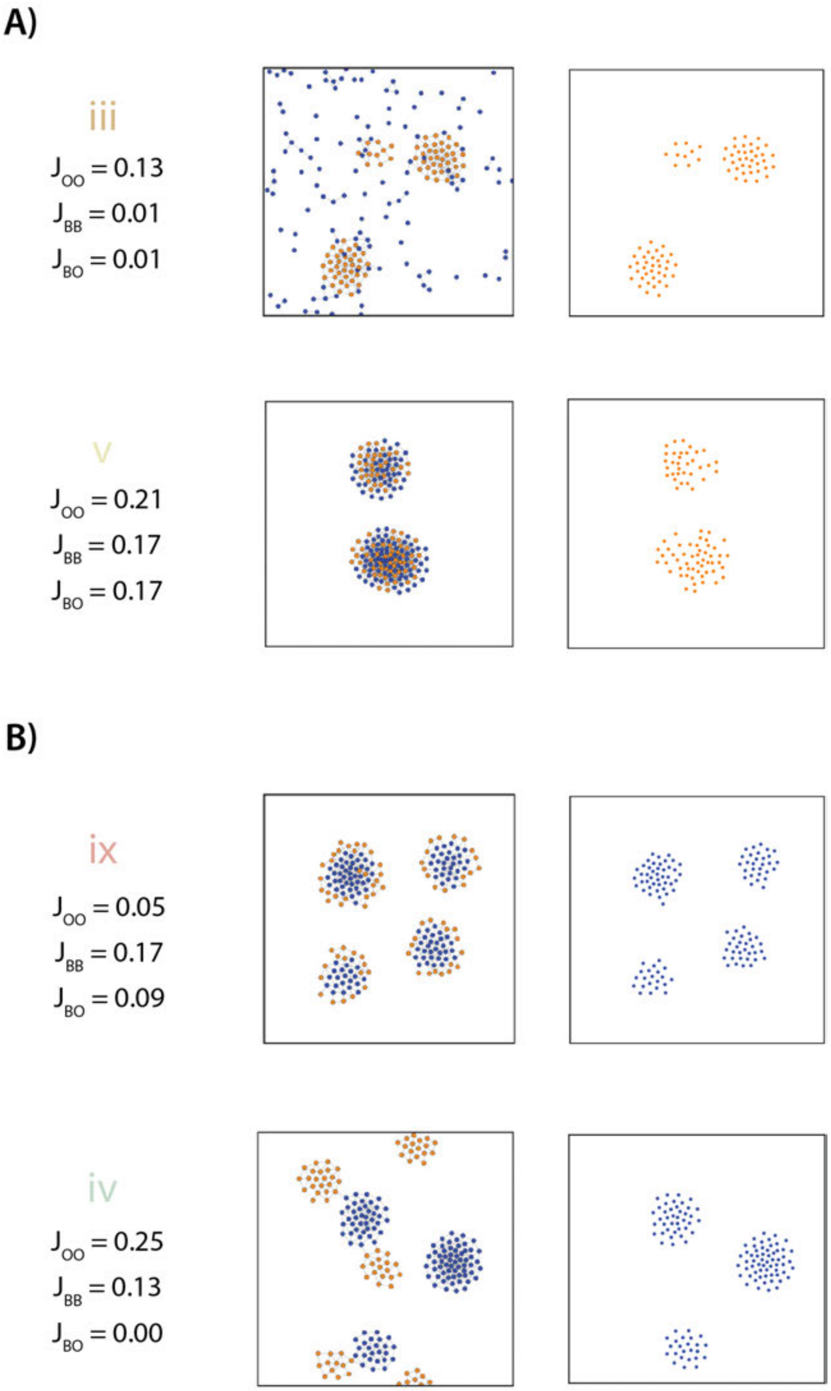

Supplementary Figure 39: **Examples of misclassified cell configurations due to similarity of single-cell topological clusters.** (A) Simulations are misclassified when only orange cell positions are considered. (B) Simulations are misclassified when only blue cell positions are considered. Note that these simulations are not misclassified in dimension 1 topology, due to the absence of topological loops.

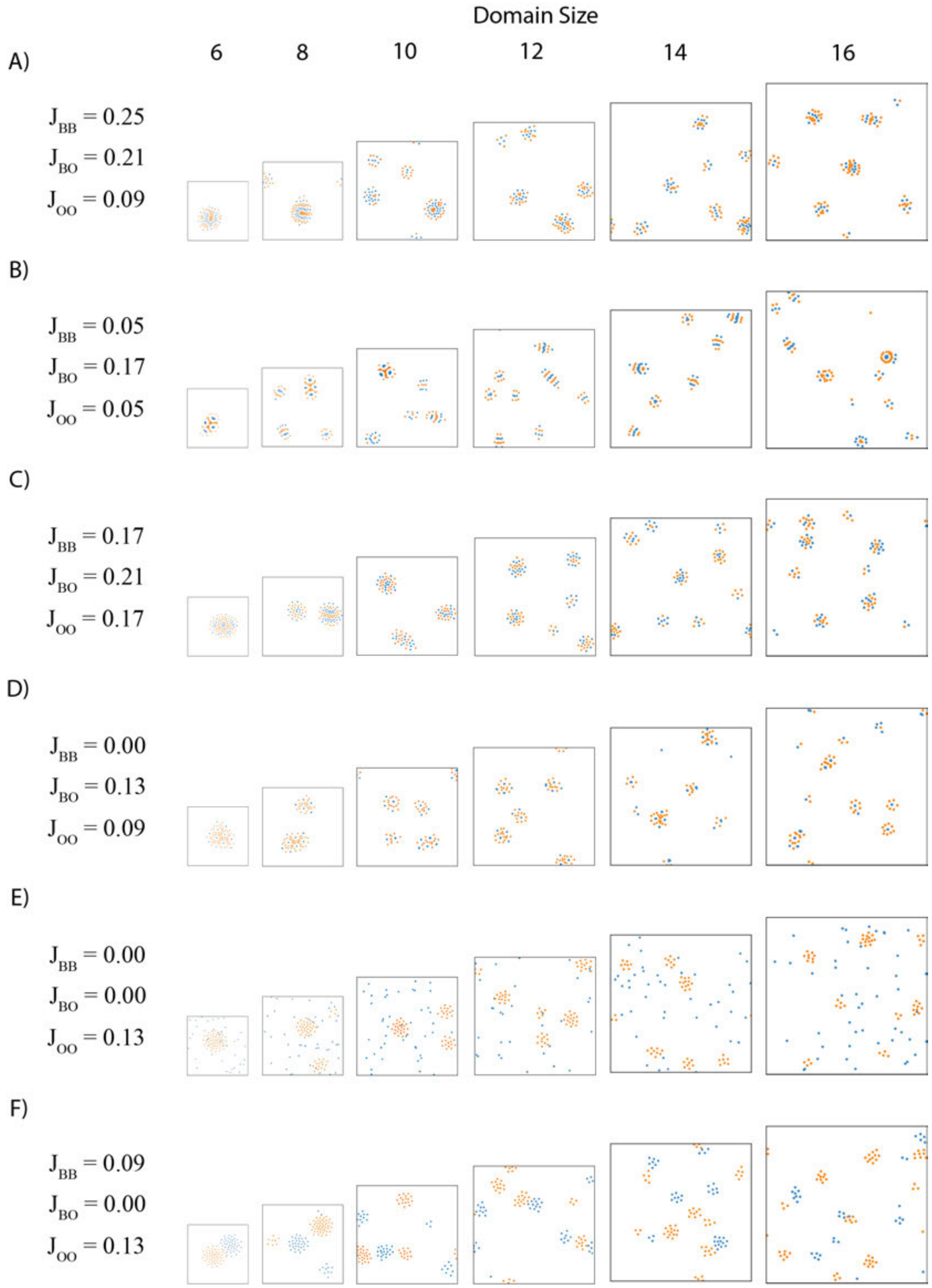

Supplementary Figure 40: **Simulations at varying domain sizes with constant population size.** (A) Orange and purple particles aggregate into clusters while blue particles remain individually dispersed. (B) Purple particles are engulfed within orange clusters. (C) Particles aggregate to form clusters due to high adhesion. Clusters contain a mixture of particle types. (D) Blue and purple particles surround the orange particles due to high blue-orange and purple-orange adhesion. (E) Orange and purple particles aggregate into clusters while blue particles remain individually dispersed. (F) Blue and orange particles aggregate into clusters due to high adhesion.

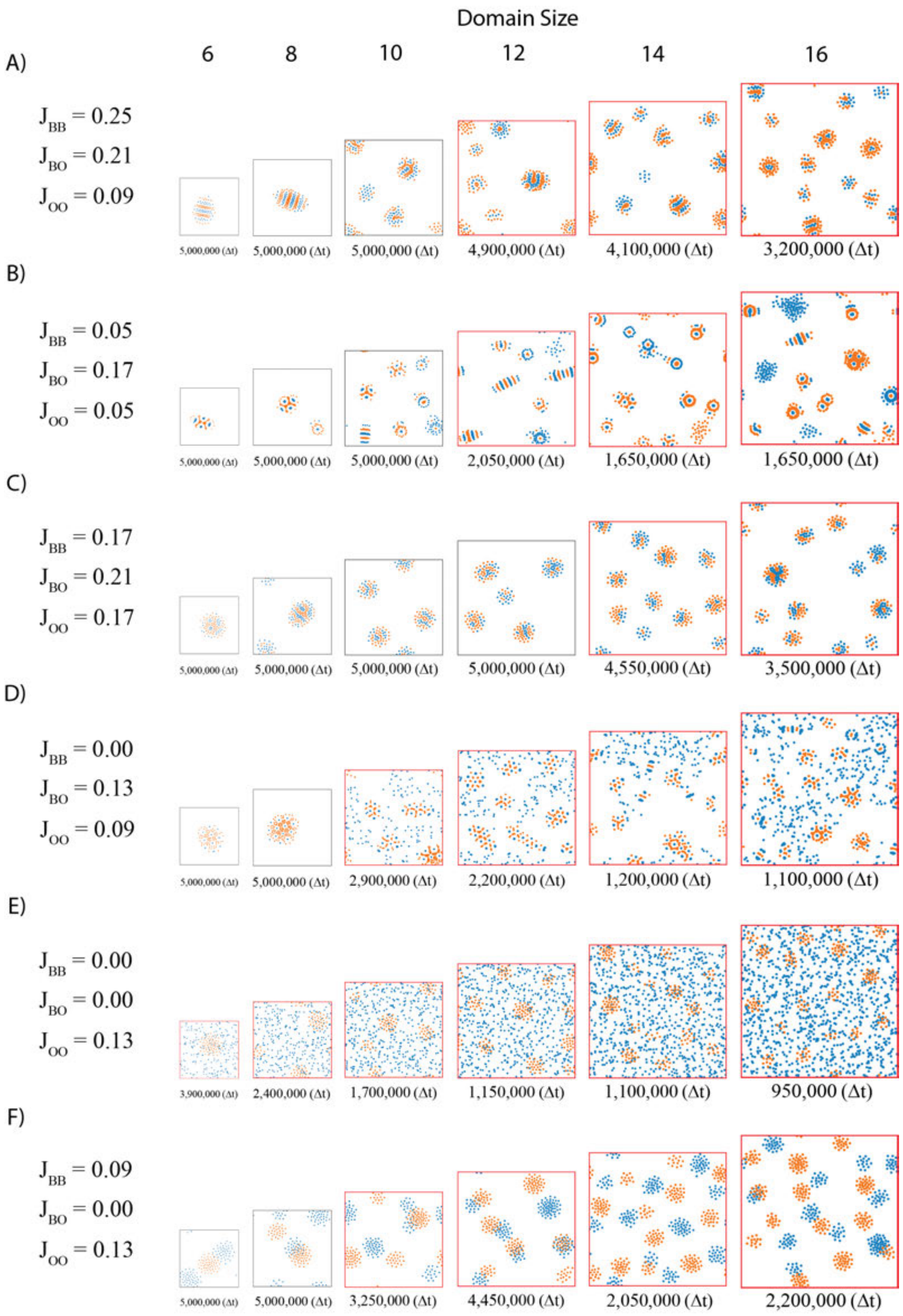

Supplementary Figure 41: **Simulations at varying domain sizes with varying population size.** (A) Orange and purple particles aggregate into clusters while blue particles remain individually dispersed. (B) Purple particles are engulfed within orange clusters. (C) Particles aggregate to form clusters due to high adhesion. Clusters contain a mixture of particle types. (D) Blue and purple particles surround the orange particles due to high blue-orange and purple-orange adhesion. (E) Orange and purple particles aggregate into clusters while blue particles remain individually dispersed. (F) Blue and orange particles aggregate into clusters due to high adhesion. Snapshots outlined in red did not run to completion in the allotted time due to the combinatorial complexity of force calculations with exponentially increasing population of orange cells.

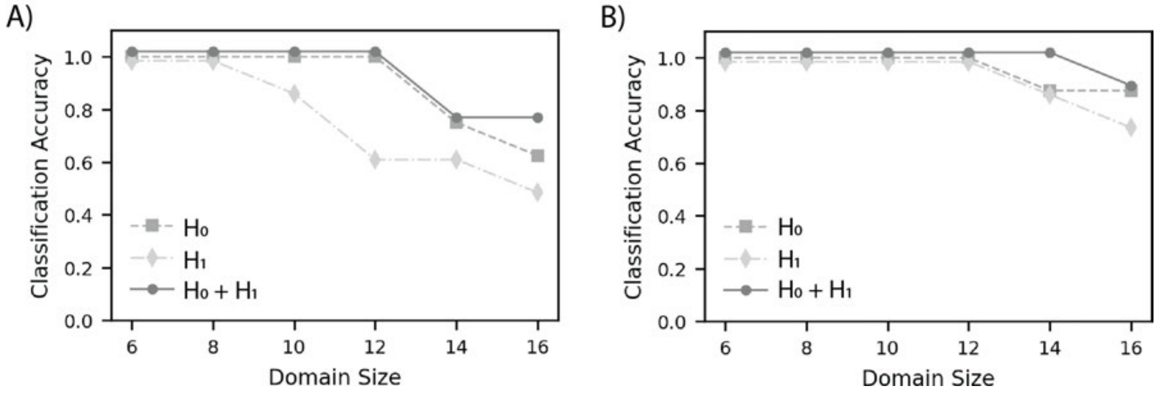

Supplementary Figure 42: **Classification accuracy of persistence images at various domain sizes.** (A) at constant population size, and (B) at varying population size.

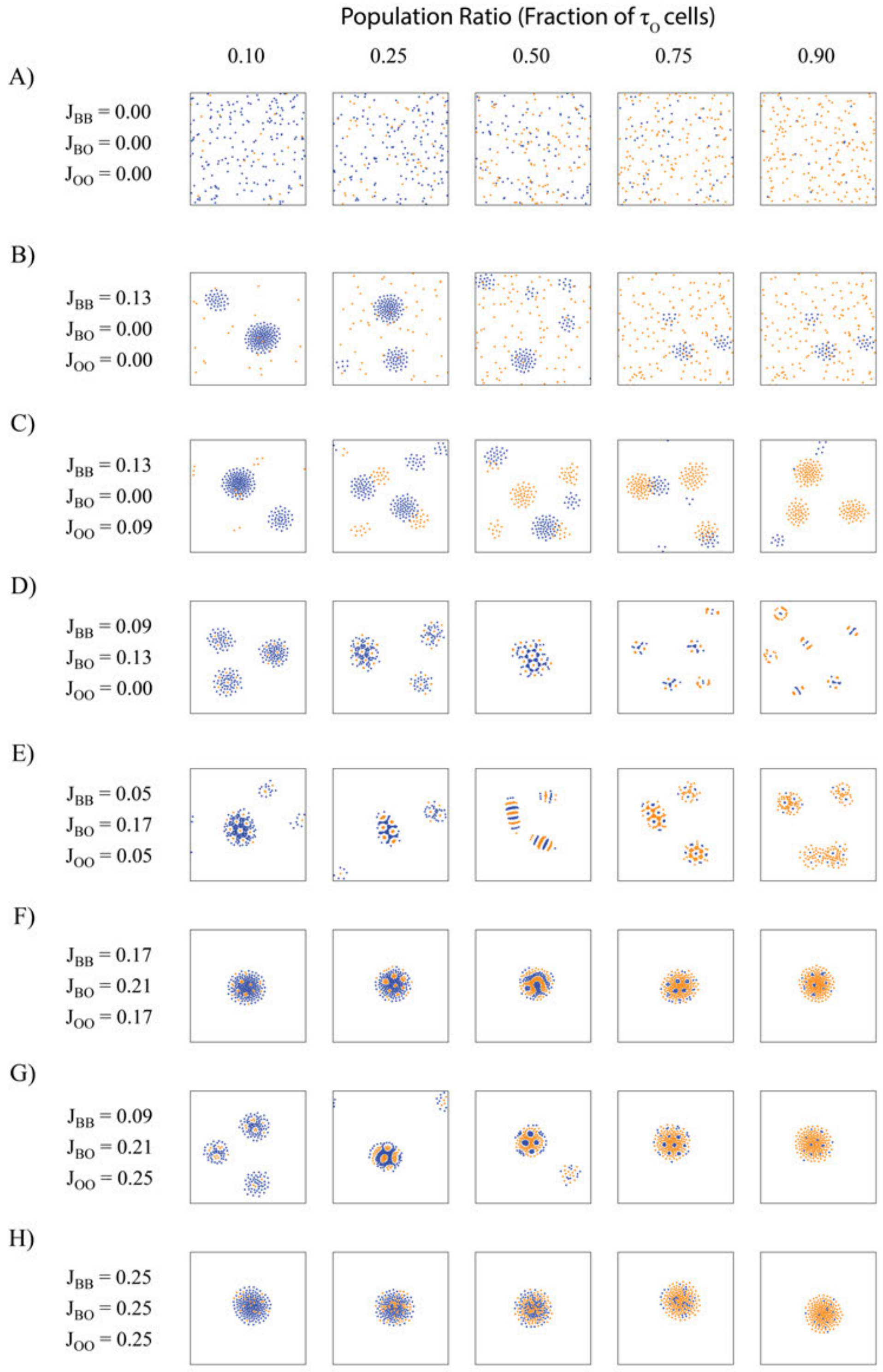

Supplementary Figure 43: **Simulation snapshots showing self-organization in a heterogeneous population at varying population ratios.** (A) Particles remain individually dispersed at low adhesion values. (B) Blue particles aggregate into clusters due to high blue-blue adhesion. (C) Complete sorting simulation where both blue and orange particles form separate clusters due to high homotypic adhesion. (D-G) High heterotypic adhesion results in configurations that maximize the interaction between the two cell types, forming hexagonal, striped and spotted patterns. (H) Well-mixed clusters are obtained when homotypic and heterotypic adhesion values are greater than zero and (approximately) equal.

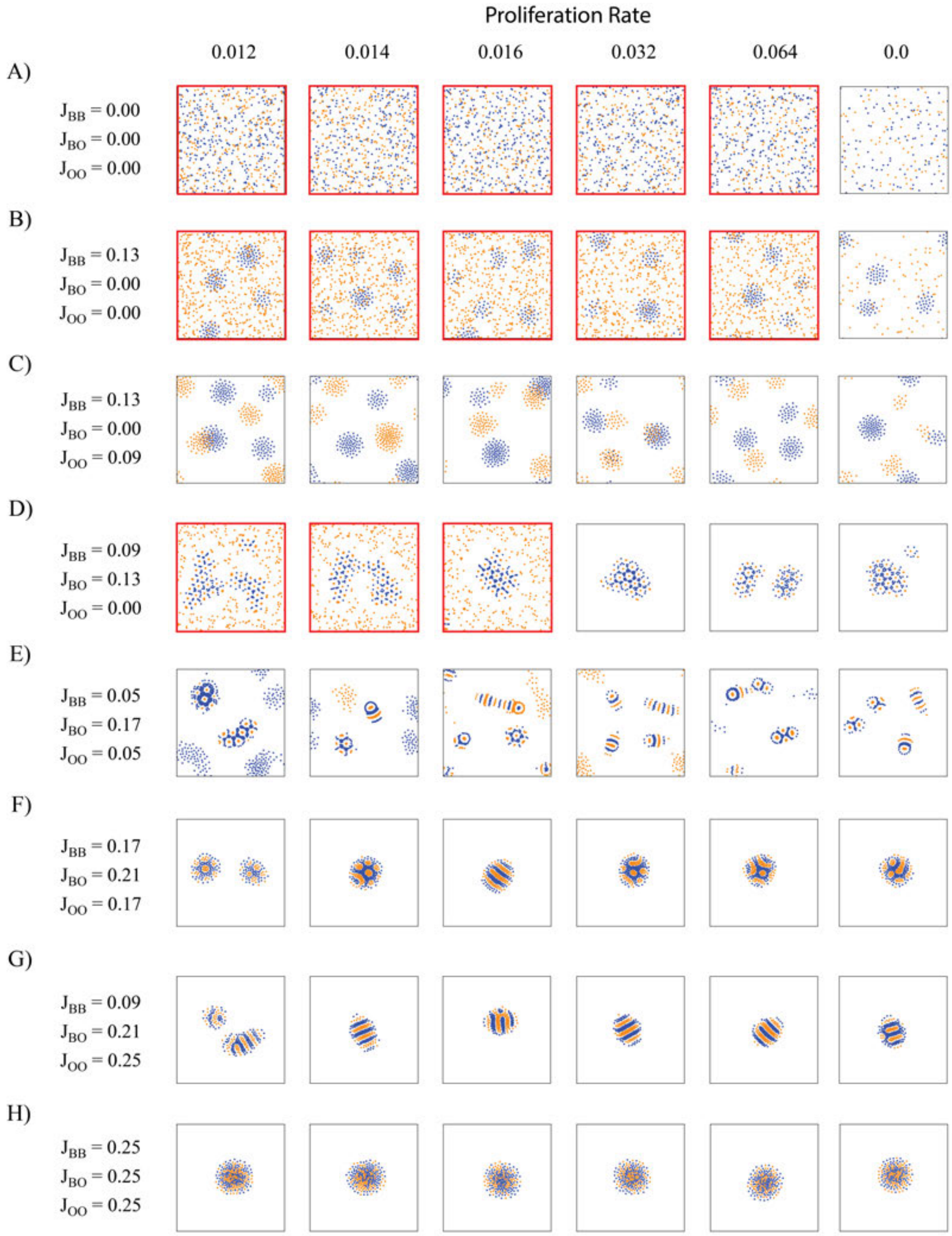

Supplementary Figure 44: **Simulation snapshots showing self-organization in a heterogeneous population at varying population size.** (A) Particles remain individually dispersed at low adhesion values. (B) Blue particles aggregate into clusters due to high blue-blue adhesion. (C) Complete sorting simulation where both blue and orange particles form separate clusters due to high homotypic adhesion. (D-G) High heterotypic adhesion results in configurations that maximize the interaction between the two cell types, forming hexagonal, striped and spotted patterns. (H) Well-mixed clusters are obtained when homotypic and heterotypic adhesion values are greater than zero and (approximately) equal. Snapshots outlined in red did not run to completion in the allotted time due to the combinatorial complexity of force calculations with exponentially increasing population of orange cells.

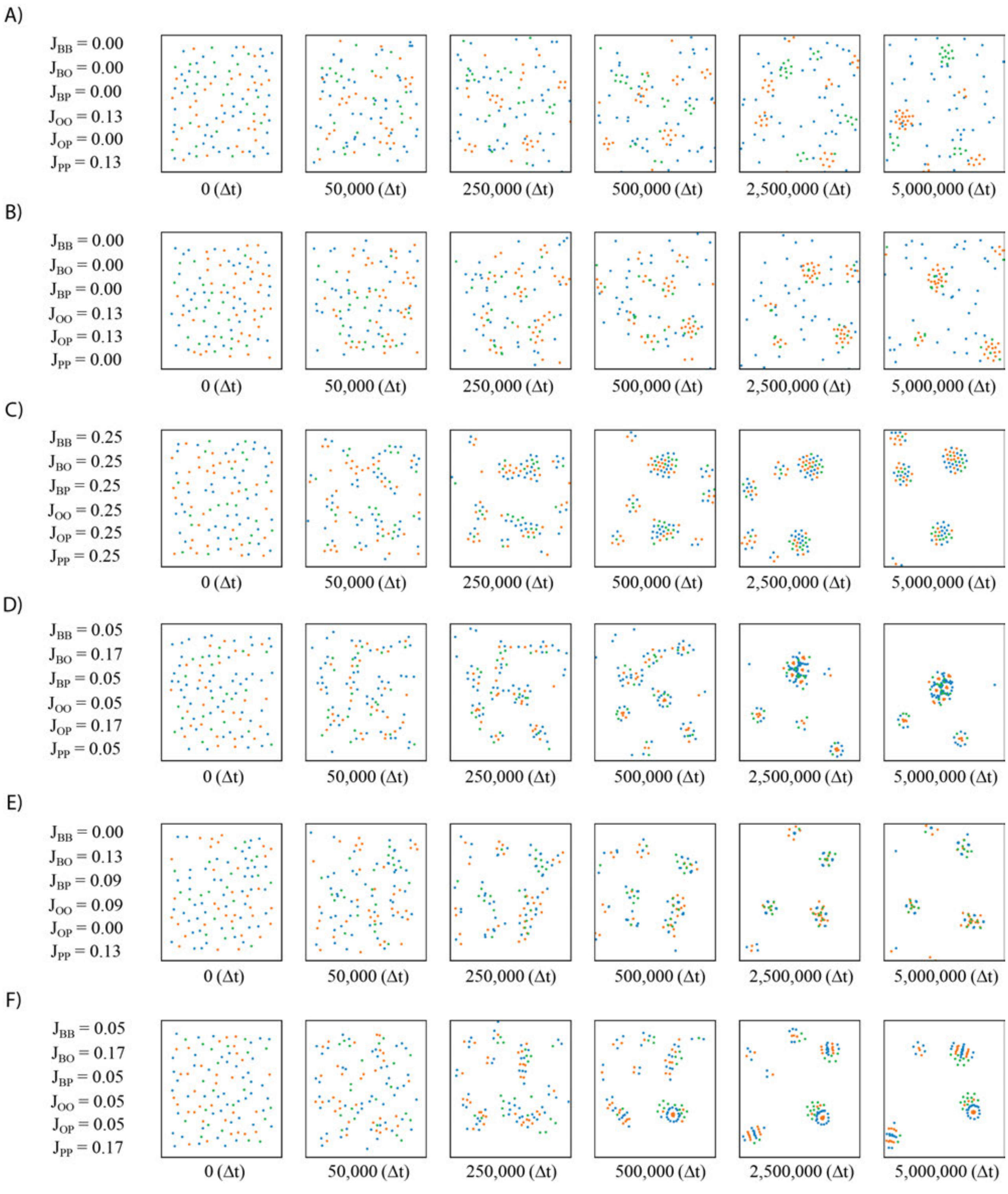

Supplementary Figure 45: **Simulation snapshots showing self-organization in a heterogeneous population with three cell types (50% orange, 30% blue, 20% green) at a constant population size of 100.** (A) Orange and green cells aggregate into separate clusters, while blue cells remain individually dispersed. (B) Green cells are engulfed within orange clusters, while blue cells remain individually dispersed. (C) Cells indiscriminately aggregate into clusters due to high adhesion. (D) Hexagonal arrangement of orange clusters surrounded by blue and green cells due to high blue-orange and green-orange adhesion. (E) Hexagonal arrangement of blue cells with offset hexagonal arrangement of orange and green cells (F) Alternating stripes of blue and orange cells with some displaced green cells.

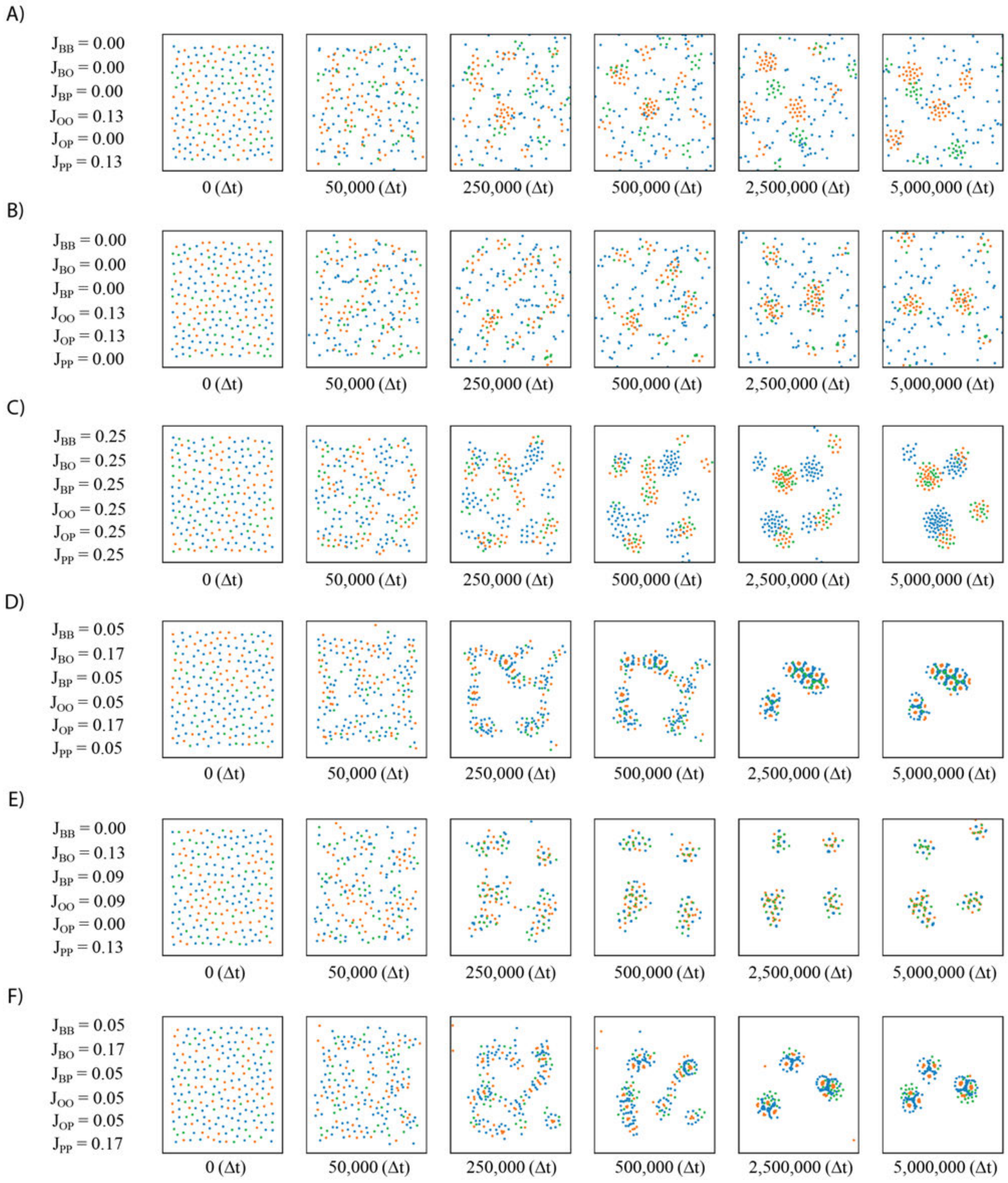

Supplementary Figure 46: **Simulation snapshots showing self-organization in a heterogeneous population with three cell types (50% orange, 30% blue, 20% green) at a constant population size of 200.** (A) Orange and green cells aggregate into separate clusters, while blue cells remain individually dispersed. (B) Green cells are engulfed within orange clusters, while blue cells remain individually dispersed. (C) Cells partially sorted within clusters due to high adhesion. (D) Hexagonal arrangement of orange clusters surrounded by blue and green cells due to high blue-orange and green-orange adhesion. (E) Hexagonal arrangement of blue cells with offset hexagonal arrangement of orange and green cells (F) Hexagonal arrangement of orange clusters surrounded by blue and green cells.

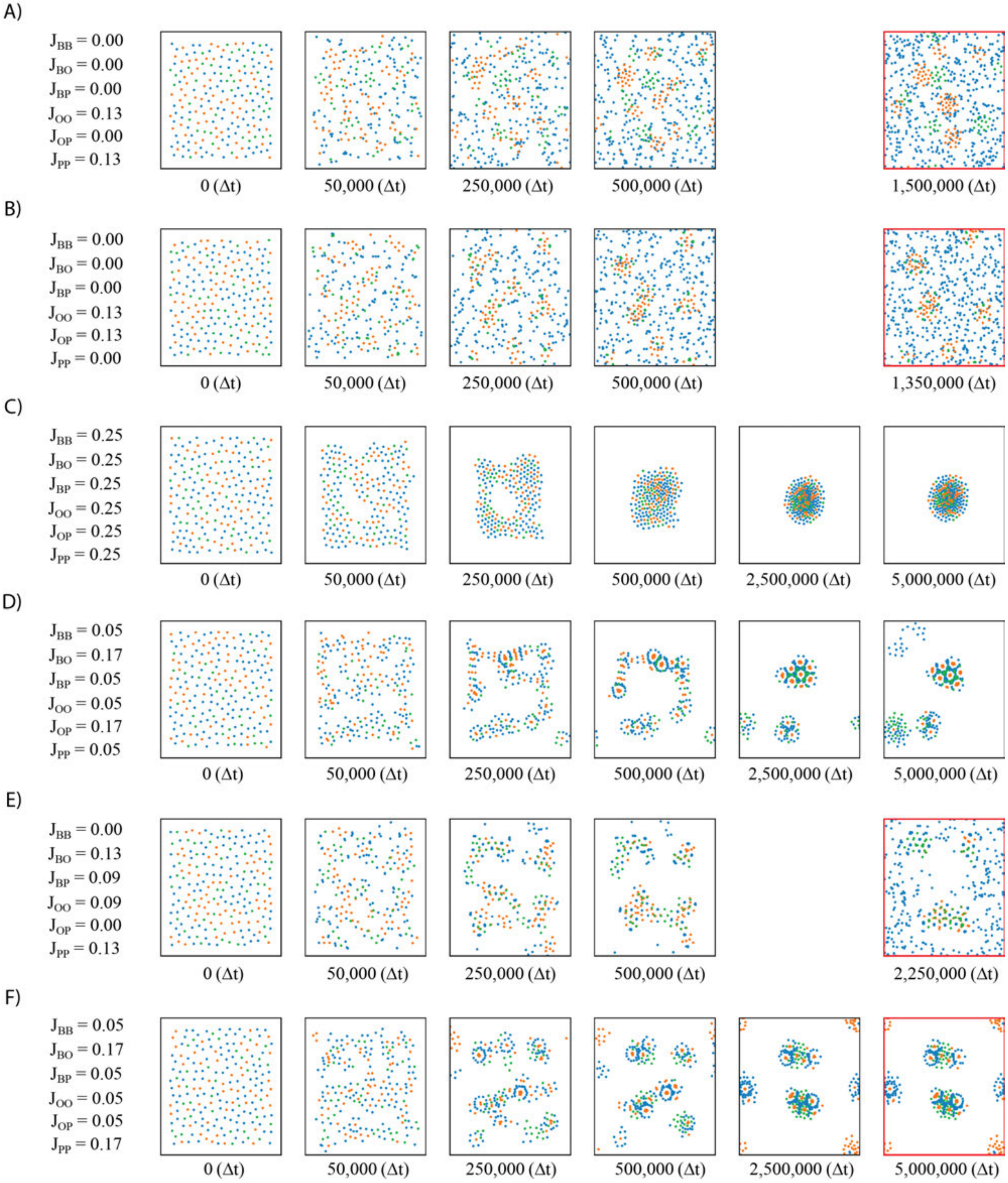

Supplementary Figure 47: **Simulation snapshots showing self-organization in a heterogeneous population with three cell types (50% orange, 30% blue, 20% green) with proliferation.** (A) Orange and green cells aggregate into separate clusters while blue cells remain individually dispersed. (B) Green cells are engulfed within orange clusters, while blue cells remain individually dispersed. (C) Cells indiscriminately aggregate into clusters due to high adhesion. Clusters contain a mixture of particle types. (D) Hexagonal arrangement of orange clusters surrounded by blue and green cells due to high blue-orange and green-orange adhesion. (E) Hexagonal arrangement of green cells with offset hexagonal arrangement of orange cells, and dispersed blue cells (F) Hexagonal arrangement of orange clusters surrounded by blue and green cells.. Snapshots outlined in red did not run to completion in the allotted time due to the combinatorial complexity of force calculations with exponentially increasing population of orange cells.

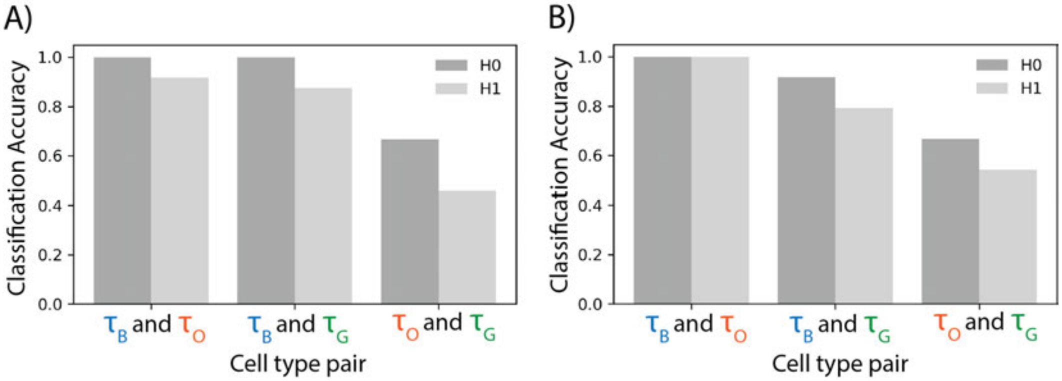

Supplementary Figure 48: **Classification accuracy of persistence images computed from different pairs of cell types.** (A) at constant population size, and (B) at varying population size.

Cell Types	Feature	Dim. Reduction	Feature Size	Classification Metrics Accuracy	Precision	Recall	F1
τ_B, τ_O	H_0	N/A	3×200	0.99	1.00	0.99	0.99
τ_B, τ_O	H_1	N/A	$3 \times 200 \times 100$	0.97	1.00	0.97	0.98
τ_B, τ_O	H_0, H_1	N/A	$3 \times 200 \times 101$	0.99	1.00	0.99	0.99
τ_B, τ_O	H_0	PHATE	3×20	0.91	1.00	0.93	0.96
τ_B, τ_O	H_1	PHATE	3×20	0.98	0.99	0.97	0.98
τ_B, τ_O	H_0, H_1	PHATE	$3 \times 20 \times 2$	0.95	1.00	0.97	0.98
τ_B, τ_O	H_0	AE	3×20	0.93	0.95	0.91	0.93
τ_B, τ_O	H_1	AE	3×20	0.97	0.94	0.89	0.91
τ_B, τ_O	H_0, H_1	AE	$3 \times 20 \times 2$	0.96	0.96	0.92	0.94
τ_B	H_0	N/A	200	0.90	0.87	0.82	0.84
τ_B	H_1	N/A	200×100	0.83	0.79	0.81	0.80
τ_B	H_0, H_1	N/A	200×101	0.92	0.89	0.84	0.86
τ_B	H_0	PHATE	20	0.79	0.80	0.72	0.76
τ_B	H_1	PHATE	20	0.72	0.75	0.74	0.74
τ_B	H_0, H_1	PHATE	20×2	0.77	0.79	0.76	0.77
τ_B	H_0	AE	20	0.86	0.88	0.83	0.85
τ_B	H_1	AE	20	0.74	0.70	0.68	0.69
τ_B	H_0, H_1	AE	20×2	0.87	0.88	0.84	0.86
τ_O	H_0	N/A	200	0.73	0.77	0.67	0.72
τ_O	H_1	N/A	200×100	0.67	0.69	0.67	0.68
τ_O	H_0, H_1	N/A	200×101	0.71	0.76	0.69	0.72
τ_O	H_0	PHATE	20	0.71	0.68	0.73	0.70
τ_O	H_1	PHATE	20	0.68	0.64	0.69	0.66
τ_O	H_0, H_1	PHATE	20×2	0.67	0.70	0.71	0.70
τ_O	H_0	AE	20	0.69	0.65	0.68	0.66
τ_O	H_1	AE	20	0.61	0.62	0.64	0.63
τ_O	H_0, H_1	AE	20×2	0.71	0.67	0.68	0.67

Supplementary Table 1: Unsupervised classification accuracy of persistence images at constant population size.

Cell Types	Feature	Dim. Reduction	Feature Size	Classification Metrics Accuracy	Precision	Recall	F1
τ_B, τ_O	H_0	N/A	3×200	0.91	0.85	0.95	0.90
τ_B, τ_O	H_1	N/A	3×200	0.83	0.77	0.79	0.78
τ_B, τ_O	H_0, H_1	N/A	$3 \times 200 \times 2$	0.93	0.91	0.93	0.92
τ_B, τ_O	H_0	PHATE	3×20	0.82	0.76	0.79	0.77
τ_B, τ_O	H_1	PHATE	3×20	0.65	0.58	0.61	0.59
τ_B, τ_O	H_0, H_1	PHATE	$3 \times 20 \times 2$	0.84	0.80	0.79	0.79
τ_B, τ_O	H_0	AE	3×20	0.86	0.86	0.88	0.87
τ_B, τ_O	H_1	AE	3×20	0.77	0.72	0.74	0.73
τ_B, τ_O	H_0, H_1	AE	$3 \times 20 \times 2$	0.86	0.84	0.82	0.83
τ_B	H_0	N/A	200	0.77	0.73	0.68	0.70
τ_B	H_1	N/A	200	0.74	0.69	0.70	0.69
τ_B	H_0, H_1	N/A	200×2	0.79	0.78	0.76	0.77
τ_B	H_0	PHATE	20	0.78	0.71	0.73	0.72
τ_B	H_1	PHATE	20	0.64	0.66	0.60	0.63
τ_B	H_0, H_1	PHATE	20×2	0.81	0.77	0.76	0.76
τ_B	H_0	AE	20	0.74	0.76	0.73	0.74
τ_B	H_1	AE	20	0.70	0.68	0.68	0.68
τ_B	H_0, H_1	AE	20×2	0.86	0.81	0.78	0.79
τ_O	H_0	N/A	200	0.66	0.58	0.62	0.60
τ_O	H_1	N/A	200	0.58	0.53	0.47	0.50
τ_O	H_0, H_1	N/A	200×2	0.65	0.60	0.62	0.61
τ_O	H_0	PHATE	20	0.60	0.57	0.62	0.59
τ_O	H_1	PHATE	20	0.53	0.51	0.45	0.48
τ_O	H_0, H_1	PHATE	20×2	0.67	0.58	0.61	0.59
τ_O	H_0	AE	20	0.64	0.60	0.59	0.59
τ_O	H_1	AE	20	0.57	0.58	0.51	0.54
τ_O	H_0, H_1	AE	20×2	0.62	0.61	0.61	0.61

Supplementary Table 2: Unsupervised classification accuracy of normalized persistence curves at constant population size.

Cell Types	Feature	Dim. Reduction	Feature Size	Classification Metrics Accuracy	Precision	Recall	F1
τ_B, τ_O	RDF	N/A	3×200	0.98	0.92	0.86	0.89
τ_B, τ_O	ADF	N/A	3×100	0.52	0.54	0.51	0.52
τ_B, τ_O	RDF, ADF	N/A	3×300	0.95	0.94	0.91	0.92
τ_B, τ_O	RDF	PHATE	3×20	0.97	0.96	0.94	0.95
τ_B, τ_O	ADF	PHATE	3×20	0.38	0.41	0.35	0.38
τ_B, τ_O	RDF, ADF	PHATE	$3 \times 20 \times 2$	0.94	0.91	0.88	0.89
τ_B, τ_O	RDF	AE	3×20	0.94	0.97	0.92	0.94
τ_B, τ_O	ADF	AE	3×20	0.47	0.41	0.36	0.38
τ_B, τ_O	RDF, ADF	AE	$3 \times 20 \times 2$	0.93	0.97	0.93	0.95
τ_B	RDF	N/A	200	0.92	0.89	0.88	0.88
τ_B	ADF	N/A	100	0.44	0.39	0.42	0.40
τ_B	RDF, ADF	N/A	300	0.88	0.93	0.90	0.91
τ_B	RDF	PHATE	20	0.93	0.88	0.89	0.88
τ_B	ADF	PHATE	20	0.37	0.35	0.29	0.31
τ_B	RDF, ADF	PHATE	20×2	0.90	0.86	0.91	0.88
τ_B	RDF	AE	20	0.88	0.83	0.84	0.83
τ_B	ADF	AE	20	0.43	0.32	0.39	0.35
τ_B	RDF, ADF	AE	20×2	0.81	0.90	0.84	0.87
τ_O	RDF	N/A	200	0.74	0.77	0.71	0.74
τ_O	ADF	N/A	100	0.38	0.38	0.34	0.36
τ_O	RDF, ADF	N/A	300	0.74	0.82	0.73	0.77
τ_O	RDF	PHATE	20	0.75	0.68	0.69	0.68
τ_O	ADF	PHATE	20	0.32	0.28	0.22	0.25
τ_O	RDF, ADF	PHATE	20×2	0.72	0.69	0.69	0.69
τ_O	RDF	AE	20	0.72	0.64	0.66	0.65
τ_O	ADF	AE	20	0.36	0.31	0.28	0.29
τ_O	RDF, ADF	AE	20×2	0.68	0.66	0.65	0.65

Supplementary Table 3: Unsupervised classification accuracy of order parameters at constant population size.

Cell Types	Feature	Dim. Reduction	Feature Size	Classification Metrics Accuracy	Precision	Recall	F1
τ_B, τ_O	H_0	N/A	3×200	0.92	1.00	0.93	0.96
τ_B, τ_O	H_1	N/A	$3 \times 200 \times 100$	0.85	0.92	0.81	0.86
τ_B, τ_O	H_0, H_1	N/A	$3 \times 200 \times 101$	0.90	1.00	0.94	0.97
τ_B, τ_O	H_0	PHATE	3×20	0.74	0.81	0.76	0.78
τ_B, τ_O	H_1	PHATE	3×20	0.52	0.62	0.63	0.62
τ_B, τ_O	H_0, H_1	PHATE	$3 \times 20 \times 2$	0.79	0.79	0.77	0.78
τ_B, τ_O	H_0	AE	3×20	0.84	0.89	0.80	0.84
τ_B, τ_O	H_1	AE	3×20	0.81	0.84	0.74	0.79
τ_B, τ_O	H_0, H_1	AE	$3 \times 20 \times 2$	0.83	0.89	0.81	0.85
τ_B	H_0	N/A	200	0.78	0.81	0.75	0.78
τ_B	H_1	N/A	200×100	0.66	0.62	0.60	0.61
τ_B	H_0, H_1	N/A	200×101	0.77	0.79	0.77	0.78
τ_B	H_0	PHATE	20	0.62	0.59	0.56	0.57
τ_B	H_1	PHATE	20	0.49	0.51	0.45	0.48
τ_B	H_0, H_1	PHATE	20×2	0.63	0.66	0.63	0.64
τ_B	H_0	AE	20	0.71	0.66	0.65	0.65
τ_B	H_1	AE	20	0.68	0.69	0.63	0.66
τ_B	H_0, H_1	AE	20×2	0.73	0.70	0.66	0.68
τ_O	H_0	N/A	200	0.64	0.68	0.61	0.64
τ_O	H_1	N/A	200×100	0.66	0.68	0.63	0.65
τ_O	H_0, H_1	N/A	200×101	0.63	0.70	0.62	0.66
τ_O	H_0	PHATE	20	0.61	0.64	0.65	0.64
τ_O	H_1	PHATE	20	0.38	0.44	0.39	0.41
τ_O	H_0, H_1	PHATE	20×2	0.59	0.64	0.67	0.65
τ_O	H_0	AE	20	0.62	0.65	0.59	0.62
τ_O	H_1	AE	20	0.57	0.61	0.62	0.61
τ_O	H_0, H_1	AE	20×2	0.63	0.64	0.62	0.63

Supplementary Table 4: Unsupervised classification accuracy of persistence images at varying population size.

Cell Types	Feature	Dim. Reduction	Feature Size	Classification Metrics Accuracy	Precision	Recall	F1
τ_B, τ_O	H_0	N/A	3×200	0.86	0.89	0.81	0.85
τ_B, τ_O	H_1	N/A	3×200	0.55	0.63	0.60	0.61
τ_B, τ_O	H_0, H_1	N/A	$3 \times 200 \times 2$	0.79	0.82	0.83	0.83
τ_B, τ_O	H_0	PHATE	3×20	0.79	0.77	0.71	0.74
τ_B, τ_O	H_1	PHATE	3×20	0.52	0.64	0.59	0.61
τ_B, τ_O	H_0, H_1	PHATE	$3 \times 20 \times 2$	0.80	0.79	0.73	0.76
τ_B, τ_O	H_0	AE	3×20	0.84	0.87	0.88	0.88
τ_B, τ_O	H_1	AE	3×20	0.49	0.60	0.56	0.58
τ_B, τ_O	H_0, H_1	AE	$3 \times 20 \times 2$	0.86	0.87	0.89	0.88
τ_B	H_0	N/A	200	0.74	0.78	0.75	0.76
τ_B	H_1	N/A	200	0.53	0.49	0.51	0.50
τ_B	H_0, H_1	N/A	200×2	0.70	0.76	0.77	0.76
τ_B	H_0	PHATE	20	0.74	0.73	0.75	0.74
τ_B	H_1	PHATE	20	0.41	0.34	0.39	0.36
τ_B	H_0, H_1	PHATE	20×2	0.73	0.74	0.74	0.74
τ_B	H_0	AE	20	0.72	0.76	0.69	0.72
τ_B	H_1	AE	20	0.49	0.42	0.51	0.46
τ_B	H_0, H_1	AE	20×2	0.76	0.78	0.71	0.74
τ_O	H_0	N/A	200	0.68	0.73	0.70	0.71
τ_O	H_1	N/A	200	0.40	0.36	0.39	0.37
τ_O	H_0, H_1	N/A	200×2	0.71	0.75	0.69	0.72
τ_O	H_0	PHATE	20	0.62	0.68	0.56	0.61
τ_O	H_1	PHATE	20	0.44	0.47	0.37	0.41
τ_O	H_0, H_1	PHATE	20×2	0.67	0.68	0.60	0.64
τ_O	H_0	AE	20	0.65	0.71	0.63	0.67
τ_O	H_1	AE	20	0.38	0.42	0.33	0.37
τ_O	H_0, H_1	AE	20×2	0.67	0.68	0.60	0.64

Supplementary Table 5: Unsupervised classification accuracy of normalized persistence curves at varying population size.

Cell Types	Feature	Dim. Reduction	Feature Size	Classification Metrics Accuracy	Precision	Recall	F1
τ_B, τ_O	RDF	N/A	3×200	0.94	0.90	0.92	0.91
τ_B, τ_O	ADF	N/A	3×100	0.45	0.53	0.44	0.48
τ_B, τ_O	RDF, ADF	N/A	3×300	0.93	0.91	0.91	0.91
τ_B, τ_O	RDF	PHATE	3×20	0.92	0.89	0.88	0.89
τ_B, τ_O	ADF	PHATE	3×20	0.36	0.41	0.39	0.40
τ_B, τ_O	RDF, ADF	PHATE	$3 \times 20 \times 2$	0.89	0.91	0.90	0.90
τ_B, τ_O	RDF	AE	3×20	0.88	0.89	0.87	0.88
τ_B, τ_O	ADF	AE	3×20	0.47	0.42	0.40	0.41
τ_B, τ_O	RDF, ADF	AE	$3 \times 20 \times 2$	0.84	0.90	0.91	0.90
τ_B	RDF	N/A	200	0.81	0.79	0.76	0.78
τ_B	ADF	N/A	100	0.46	0.47	0.42	0.44
τ_B	RDF, ADF	N/A	300	0.77	0.77	0.78	0.78
τ_B	RDF	PHATE	20	0.70	0.67	0.68	0.68
τ_B	ADF	PHATE	20	0.39	0.44	0.41	0.42
τ_B	RDF, ADF	PHATE	20×2	0.64	0.70	0.67	0.68
τ_B	RDF	AE	20	0.74	0.76	0.70	0.73
τ_B	ADF	AE	20	0.43	0.38	0.41	0.39
τ_B	RDF, ADF	AE	20×2	0.73	0.74	0.72	0.73
τ_O	RDF	N/A	200	0.69	0.64	0.67	0.65
τ_O	ADF	N/A	100	0.43	0.37	0.39	0.38
τ_O	RDF, ADF	N/A	300	0.76	0.66	0.65	0.65
τ_O	RDF	PHATE	20	0.72	0.68	0.72	0.70
τ_O	ADF	PHATE	20	0.41	0.38	0.45	0.41
τ_O	RDF, ADF	PHATE	20×2	0.72	0.70	0.71	0.71
τ_O	RDF	AE	20	0.66	0.68	0.64	0.66
τ_O	ADF	AE	20	0.39	0.41	0.36	0.38
τ_O	RDF, ADF	AE	20×2	0.68	0.70	0.63	0.66

Supplementary Table 6: Unsupervised classification accuracy of order parameters at varying population size.

Supplementary Note 1: We also considered classification using a softer probabilistic algorithm. Briefly, we used a soft margin support vector machine (SVM) where the soft-margin classifier:

$$\min \frac{1}{2}\|w\|^2 + C\sum_{i=1}^{n} \zeta_i$$
$$\text{subject to} \quad y_i(w^T x_i + b) \geq 1 - \zeta_i \quad \forall\, i = 1, \cdots, n, \quad \zeta_i \geq 0$$

trained using the hinge-loss function, $\max\{0, 1 - y_i(w^T x_i + b)\}$, allows flexibility for misclassifications, via slack variables ζ_i. The regularization parameter, C, controls the trade-off between maximizing the margin at the decision boundary and minimizing the loss. We trained the soft margin classifier, using the radial basis function for nonlinear transformation of the input, at various values of C and computed the accuracy using 5-fold cross-validation. Crucially, we modified the scoring function to ignore misclassification between adjacent phases in the accuracy computation.

Cell Types	Feature	Dim. Reduction	Feature Size	Classification Accuracy		
				$C = 0.5$	$C = 1.0$	$C = 2.0$
τ_B, τ_O	H_0	N/A	3 × 200	1.00	0.98	0.92
τ_B, τ_O	H_1	N/A	3 × 200 × 100	0.96	0.92	0.89
τ_B, τ_O	H_0, H_1	N/A	3 × 200 × 101	1.00	0.97	0.92
τ_B, τ_O	H_0	PHATE	3 × 20	1.00	0.90	0.88
τ_B, τ_O	H_1	PHATE	3 × 20	0.93	0.87	0.81
τ_B, τ_O	H_0, H_1	PHATE	3 × 20 × 2	1.00	0.89	0.87
τ_B, τ_O	H_0	AE	3 × 20	1.00	0.90	0.86
τ_B, τ_O	H_1	AE	3 × 20	0.95	0.88	0.81
τ_B, τ_O	H_0, H_1	AE	3 × 20 × 2	1.00	0.90	0.86

Supplementary Table 7: Soft margin SVM classification accuracy of persistence images at constant population size.

**Cell Types**	**Feature**	**Dim. Reduction**	**Feature Size**	**Classification Accuracy**		
				$C = 0.5$	$C = 1.0$	$C = 2.0$
τ_B, τ_O	H_0	N/A	3×200	0.97	0.93	0.86
τ_B, τ_O	H_1	N/A	$3 \times 200 \times 100$	0.85	0.79	0.74
τ_B, τ_O	H_0, H_1	N/A	$3 \times 200 \times 101$	0.94	0.91	0.87
τ_B, τ_O	H_0	PHATE	3×20	0.91	0.89	0.88
τ_B, τ_O	H_1	PHATE	3×20	0.82	0.79	0.76
τ_B, τ_O	H_0, H_1	PHATE	$3 \times 20 \times 2$	0.91	0.86	0.87
τ_B, τ_O	H_0	AE	3×20	0.79	0.75	0.71
τ_B, τ_O	H_1	AE	3×20	0.63	0.61	0.58
τ_B, τ_O	H_0, H_1	AE	$3 \times 20 \times 2$	0.80	0.73	0.69

Supplementary Table 8: Soft margin SVM classification accuracy of persistence images at varying population size.
